# *Bacillus cereus* cereolysin O induces pyroptosis in an undecapeptide-dependent manner

**DOI:** 10.1038/s41420-024-01887-7

**Published:** 2024-03-08

**Authors:** Yujian Wang, Jingchang Luo, Xiaolu Guan, Yan Zhao, Li Sun

**Affiliations:** 1 CAS and Shandong Province Key Laboratory of Experimental Marine Biology, Institute of Oceanology, CAS Center for Ocean Mega-Science, Chinese Academy of Sciences, Qingdao, China; 2 Laboratory for Marine Biology and Biotechnology, Qingdao Marine Science and Technology Center, Qingdao, China; 3 College of Marine Sciences, University of Chinese Academy of Sciences, Qingdao, China; 4 Tsinghua-Peking Joint Center for Life Sciences, School of Medicine, Tsinghua University, Beijing, China

**Keywords:** Cell death and immune response, Water microbiology

## Abstract

*Bacillus cereus* is a clinically significant foodborne pathogen that causes severe gastrointestinal and non-gastrointestinal disease. Cereolysin O (CLO) is a putative virulence factor of *B. cereus*, and its function remains to be investigated. In this study, we examined the biological activity of CLO from a deep sea *B. cereus* isolate. CLO was highly toxic to mammalian cells and triggered pyroptosis through NLRP3 inflammasome-mediated caspase 1 and gasdermin D activation. CLO-induced cell death involved ROS accumulation and K^+^ efflux, and was blocked by serum lipids. CLO bound specifically to cholesterol, and this binding was essential to CLO cytotoxicity. The structural integrity of the three tryptophan residues in the C-terminal undecapeptide was vital for CLO to interact with membrane lipids and cause membrane perforation. Taken together, these results provided new insights into the molecular mechanism of *B. cereus* CLO-mediated cytotoxicity.

## Introduction

*Bacillus cereus* is a Gram-positive bacterium and a foodborne pathogen that is able to cause serious human diseases. It is widespread in natural environments, including deep sea [1, 2]. The pathogenesis of *B. cereus* still remains to be explored. It has been reported that *B. cereus* produces abundant potential virulence factors, including hemolysins, phospholipases, and proteases [3, 4]. The heat-stable toxin cereulide can cause vomiting, while the three enterotoxins, i.e., the tripartite non-hemolytic enterotoxin (NHE), hemolysin BL (HBL), and the single cytotoxin K (CytK), induce diarrhoeal syndromes [5–7]. In immunocompromised patients, *B. cereus* can cause certain fatal non-gastrointestinal infections, including endophthalmitis, respiratory and urinary tract infections, endocarditis, systemic bacterial septicemia, and central nervous system infection [8–10]. Recent studies showed that HBL, NHE, and CytK could activate the NLRP3 inflammasome, which triggered pyroptosis and led to rapid death of mice [5–7]. The biological activity of other enterotoxin candidates remains to be explored.

The cholesterol-dependent cytolysin (CDC) is a family of pore-forming exotoxins traditionally thought to bind cholesterol in cell membranes and form transmembrane pores [11–13]. The pore formation involves the assembly of oligomeric and soluble monomers into annular pre-pores that undergo conformational changes to insert into the membrane, eventually forming a large amphipathic transmembrane β-barrel structure [14–16]. Cereolysin O (CLO), also known as hemolysin I, is a protein secreted by *B. cereus*. It shares 57–68% amino acid identities with the perfringolysin O (PFO) and streptolysin O of *Clostridium perfringens* and *Streptococcus pyogenes* [17, 18], and therefore is considered to belong to the CDC family. Reports showed that CLO as low as 1–2 μg administered intravenously into mice was lethal, and that CLO was able to induce the release of lactate dehydrogenase (LDH), which exacerbated *B. cereus*-induced endophthalmitis [19, 20]. In addition, recent studies showed that membrane perforation caused by CDC could activate the NOD-like receptor family pyrin domain containing 3 (NLRP3) inflammasome, which in turn induced the secretion of pro-inflammatory cytokines such as interleukin (IL)-1β and IL-18 [13, 21, 22].

Pyroptosis is a type of programmed cell death that can be caused by pathogen invasion [23]. Pyroptosis is executed by a group of pore-forming proteins called gasdermin (GSDM). In canonical and noncanonical pyroptotic pathways, caspase (Casp) 1 and Casp4/5/11, respectively, cleave gasdermin D (GSDMD) to release the N-terminal domain (GSDMD-N) [24–26]. The released GSDMD-N binds to acidic phospholipids on the plasma membrane and forms oligomeric pores that lead to cell swelling and rupture [27–29]. The GSDMD pores enables the release of large amounts of pro-inflammatory factors, notably IL-1β and IL-18, which activate macrophages and T lymphocytes and subsequently induce inflammation [30]. The NLRP3 inflammasome is critical for the activation of the Casp1-dependent canonical pathway. The NLRP3 inflammasome assembly and activation can be triggered by multiple molecular and cellular events, including pathogen infection, mitochondrial dysfunction, ROS release, lysosomal disruption, trans-Golgi disassembly, and ion flux, such as K^+^ efflux, Na^+^ influx, and Ca^2+^ mobilization [31–37].

Recently, we reported a pathogenic *B. cereus* (MB1) isolated from the deep sea in Mariana Trench [38]. MB1 exhibited strong cytotoxicity and induced pyroptosis characterized by activation of Casp1 and GSDMD and secretion of IL-1β and IL-18 [38]. Genome sequencing showed that MB1 encoded CLO, but the function of CLO is unknown. In this study, we examined the biological activity of CLO from MB1. We found that CLO was a cytotoxin that bound plasma cholesterol and induced GSDMD-mediated pyroptosis in a structure-dependent manner. Our findings added new insights into the pathogenesis of *B. cereus*.

## Results

### CLO induces pyroptosis

**Fig. 1:**
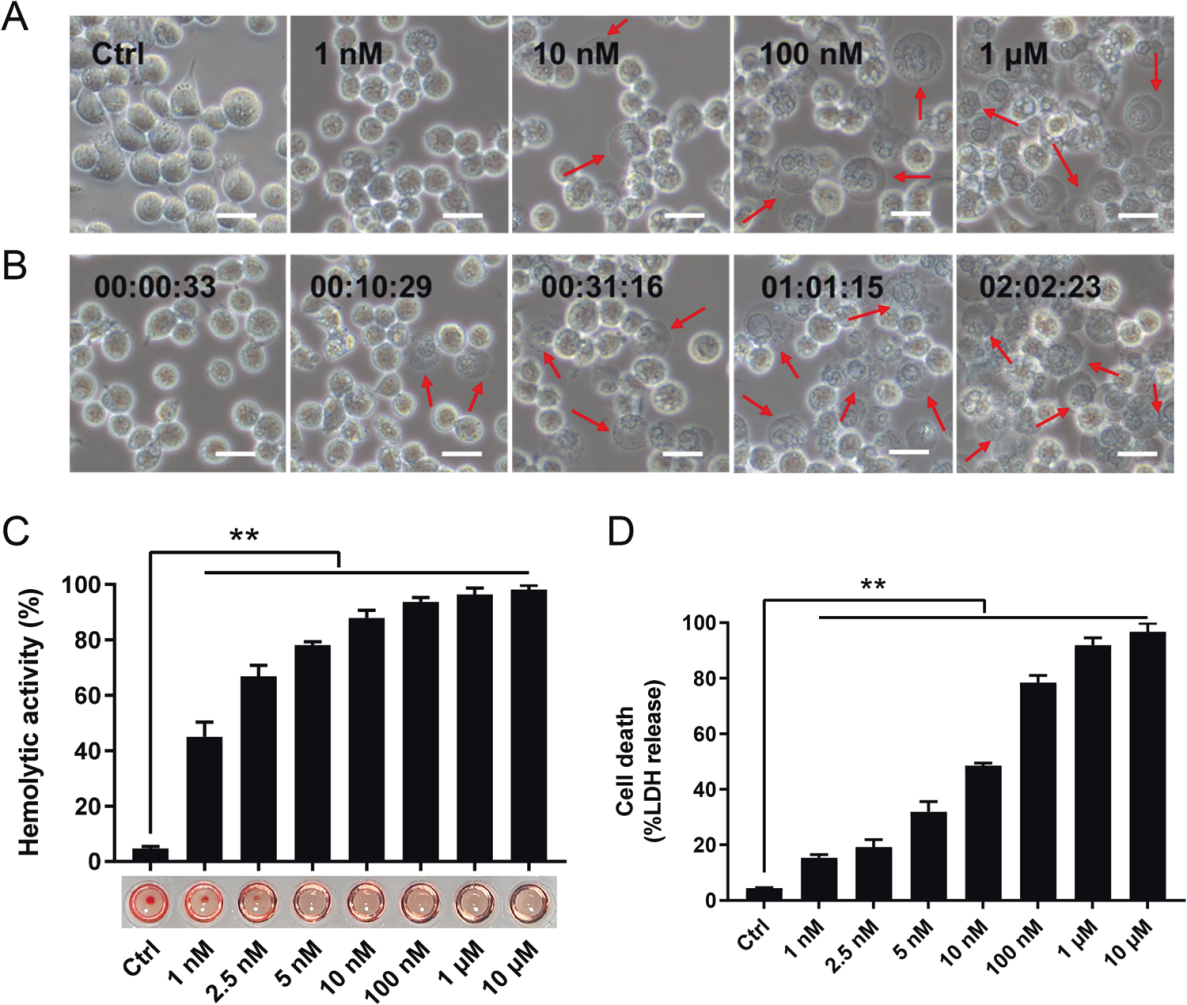
Cell death induced by CLO. **A** THP-1 cells were incubated with or without (Ctrl) CLO at different doses for 1 h and then observed with a microscope. **B** The time-lapse images of THP-1 cells treated with CLO (100 nM). **C** The hemolytic activity of CLO at different doses was determined. **D** THP-1 cells were incubated with or without (Ctrl) CLO at different doses for 1 h, and then measured for LDH release. In panels **A** and **B**, arrows indicate membrane blebbing, and the scale bar is 30 μm. In panels **C** and **D**, values are shown as means ± SD (*N* = 3). N, the number of replicates. **p < 0.01(one-way ANOVA).

**Fig. 2:**
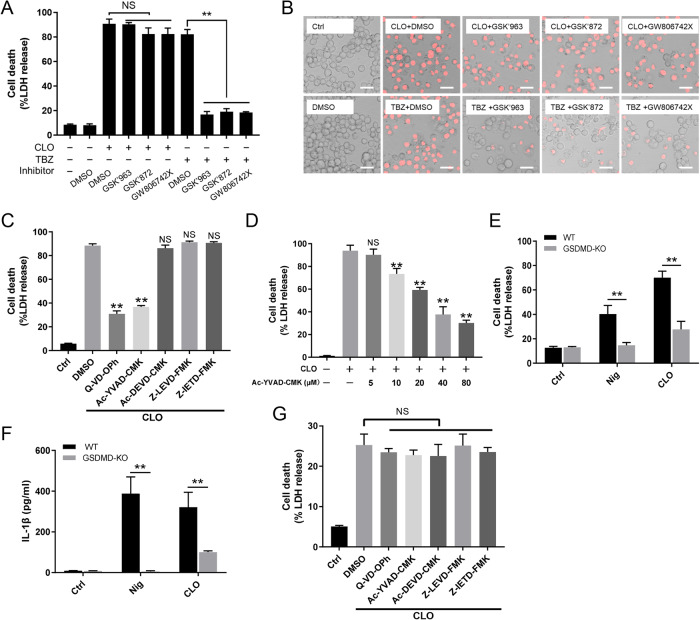
The involvement of caspase 1 and GSDMD in CLO-induced cell death. **A**, **B** THP-1 cells were treated with CLO (100 nM) or necroptosis inducer TBZ (TNFα, the SMAC mimetic BV-6 and Z-VAD) in the presence of DMSO or the inhibitors GSK’963, GSK’872, and GW806742X (targeting RIPK1, RIPK3, and MLKL respectively) for 1 h or 16 h. The cells were then subjected to LDH release determination (**A**) and microscopy after PI staining (**B**). Scale bar, 30 μm. **C** THP-1 cells were treated with or without (Ctrl) CLO (100 nM) in the presence of Q-VD-OPh, Ac-YVAD-CMK, Ac-DEVD-CMK, Z-LEVD-FMK, Z-IETD-FMK, or DMSO for 1 h. LDH release was then measured. **D** THP-1 cells were treated with or without (Ctrl) CLO (100 nM) in the presence or absence of different concentrations of Ac-YVAD-CMK for 1 h. LDH release was then measured. **E**, **F** THP-1 WT and THP-1 GSDMD-KO cells were treated with or without (Ctrl) CLO (100 nM) or nigericin (Nig) for 1 h. LDH (**E**) and IL-1β (**F**) release was then determined. **G** THP-1 GSDMD-KO cells were treated as **C** and then measured for LDH release. **p < 0.01. NS no significance (one-way ANOVA test **A**, **C**, **D**, and **G** or student’s unpaired t test **E** and **F**. Values are shown as means ± SD (*N* = 3). N the number of replicates.

Sequence analysis showed that the CLO encoded by *B. cereus* MB1 highly resembles the CLO of clinically isolated *B. cereus* group strains, with identities exceeding 90% (Supplementary Table S1). When incubated with THP-1 cells, purified recombinant CLO (Supplementary Fig. S1) caused cellular swelling in a dose- and time-dependent manner (Fig. 1A, B). CLO at 1 nM could induce marked hemolysis and LDH release (Fig. 1C, D). Similar cell death was observed with CLO-treated J774A.1 cells (Supplementary Fig. S2). CLO-induced cell death was not affected by the inhibitors targeting the key necroptosis factors, i.e., RIPK1, the RIPK3, and MLKL (Fig. 2A, B), suggesting that necroptosis was not involved in this process. In contrast, when cells were incubated with CLO in the presence of the pan-caspase inhibitor Q-VD-OPh or the Casp1 inhibitor Ac-YVAD-CMK, cell death was significantly blocked in an inhibitor dose dependent manner (Fig. 2C, D; Supplementary Fig. S3A, B). To examine whether GSDMD was required in CLO-induced cell death, the effect of CLO on GSDMD-knockout THP-1 cells (THP-1 GSDMD-KO) was determined. The results showed that compared with CLO-treated control cells, CLO-treated THP-1 GSDMD-KO cells exhibited sharply reduced release of LDH and IL-1β, and the presence of caspase inhibitors had no significant effect on the LDH release in CLO-treated GSDMD-KO cells (Fig. 2E, F, G). Time-dependent analysis showed that with the increase of time, apparent LDH release was observed in CLO-treated THP-1 Casp1 knockdown cells (THP-1 Casp1-KD) and GSDMD-KO cells, although in significantly lower amounts than that in the wild type cells (Supplementary Fig. S3C), indicating that, after prolonged interaction with the cells, CLO could induce cell death in a Casp1- and GSDMD-independent manner.

### NLRP3, ROS, and K^+^ efflux are involved in CLO-induced pyroptosis

**Fig. 3:**
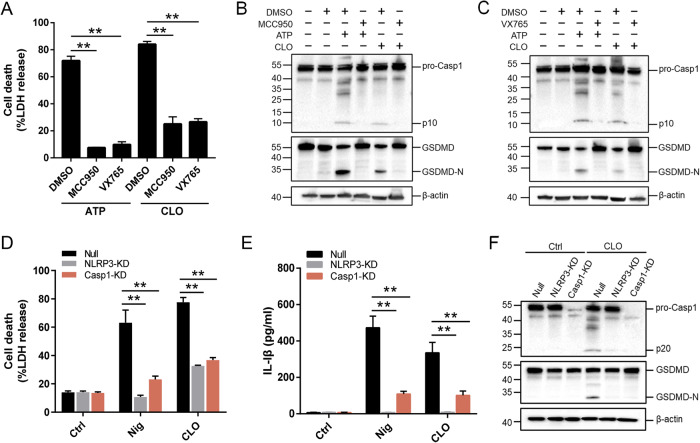
The involvement of the NLRP3-caspase 1 pathway in CLO-induced cell death. **A** J774A.1 cells were pretreated with MCC950, VX765, or DMSO for 1 h and then treated with CLO (CLO 100 nM) or ATP for 1 h. LDH release was then determined. **B**, **C** The cell lysate and supernatants from J774A.1 cells treated as above was immunoblotted with antibodies against Casp1, GSDMD, or β-actin (loading control). **D**, **E** PMA-differentiated THP-1 cells with or without (Null) deficiency in NLRP3 (NLRP3-KD) or Casp1 (Casp1-KD) were treated with or without (Ctrl) CLO (CLO 100 nM) or nigericin (Nig) for 1 h. LDH (**D**) and IL-1β (**E**) release was then determined. **F** The supernatant and the corresponding cell lysate from the above (**D**, **E**) treated J774A.1 cells were blotted with antibodies against Casp1, GSDMD, or β-actin (loading control). For panels **A**, **D**, and **E**, values are shown as means ± SD (*N* = 3). N, the number of replicates. **p < 0.01 (one-way ANOVA).

**Fig. 4:**
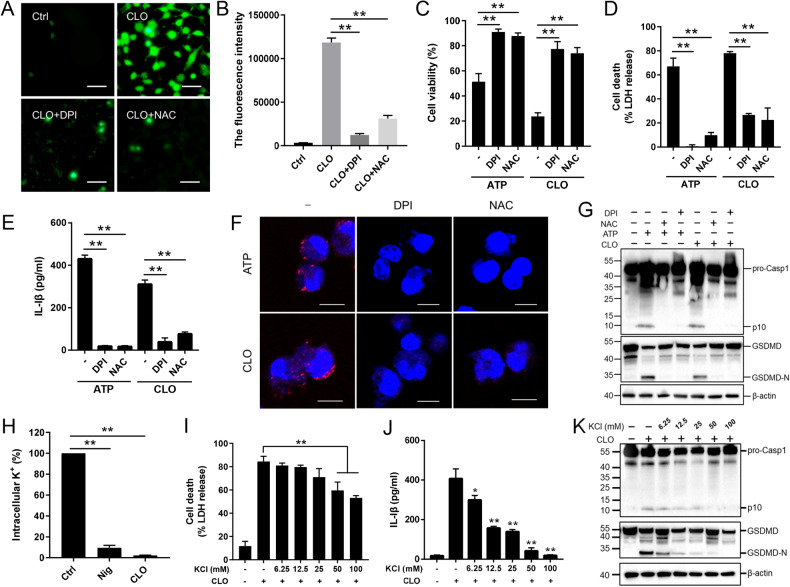
The involvement of ROS production and K^+^ efflux in CLO-induced pyroptosis. **A**, **B** J774A.1 cells pretreated with DCFH-DA were incubated with or without (Ctrl) DPI or NAC for 1 h. The cells were treated with or without CLO (10 nM) for 30 min. ROS production (**A**) and fluorescence intensity (λex, 488 nm; λem, 525 nm) (**B**) were then determined. **C**–**G** J774A.1 cells were pretreated with or without (−) DPI or NAC for 1 h and then treated with CLO (10 nM) or ATP for 1 h. Cell viability (**C**), LDH release (**D**), and IL-1β (**E**) release were determined. ASC speck (red) was detected by treating the cells with ASC-antibody and DAPI (**F**). The supernatant plus the corresponding cell lysate were blotted with antibody against Casp1, GSDMD, or β-actin (loading control) (**G**). **H** J774A.1 cells were incubated with or without (Ctrl) CLO (100 nM) or nigericin (Nig) for 30 min, and intracellular K^+^ was then determined. **I**–**K** J774A.1 cells were pretreated with or without (−) different concentrations of KCl for 1 h, and then treated with or without (−) CLO (100 nM) for 1 h. LDH (**I**) and IL-1β release (**J**) was then determined. Immunoblot analysis of Casp-1, GSDMD, or β-actin was performed as above (**K**). Scale bars of panels **A** and **F** are 30 μm and 10 μm, respectively. For panels **B**–**E** and **H**–**J**, values are shown as means ± SD (*N* = 3). N, the number of replicates. **p < 0.01, *p < 0.05. NS no significance (one-way ANOVA).

The NLRP3 inflammasome is an important regulator of Casp1 activation. In the presence of the NLRP3 inhibitor (MCC950), the ability of CLO to induce LDH release and Casp1/GSDMD activation cleavage was abolished (Fig. 3A–C). Similar abolishing effects on CLO cytotoxicity were observed with the Casp1 inhibitor (VX765) (Fig. 3A–C). In THP-1 cells with NLRP3 knockdown (NLRP3-KD) or Casp1-KD, CLO-induced LDH/IL-1β release and Casp1/GSDMD activation were significantly reduced comparing to that in the THP-1 Null cells (Fig. 3D–F). Previous studies showed that some NLRP3 inflammasome stimulators induced the production of ROS, which acted as a second messenger to drive inflammasome activation [39]. In CLO-treated J774 A.1 cells, ROS induction was observed, and this induction was markedly inhibited by ROS production inhibitor (DPI) and ROS scavenger (NAC) (Fig. 4A, B). In the presence of DPI or NAC, the viability of CLO-treated cells increased significantly (Fig. 4C), and CLO-induced LDH/IL-1β release, ASC speck formation, and Casp1/GSDMD activation significantly decreased (Fig. 4D–G; Supplementary Fig. S4A). In addition to ROS, K^+^ efflux is also a factor involved in NLRP3 inflammasome activation. In CLO-treated J774A.1 cells, the intracellular K^+^ level was significantly reduced (Fig. 4H). With the supplementation of extracellular K^+^, CLO-induced cell death, IL-1β release, and GSDMD activation were inhibited in a manner that depended on the dose of the supplemented K^+^ (Fig. 4I–K). In contrast, CLO-induced death of NLRP3-KD cells and Casp1-KD cells was not significantly affected by supplementation of extracellular K^+^ (Supplementary Fig. S4B, C).

### Plasma cholesterol binding is required for the cytotoxicity of CLO

**Fig. 5:**
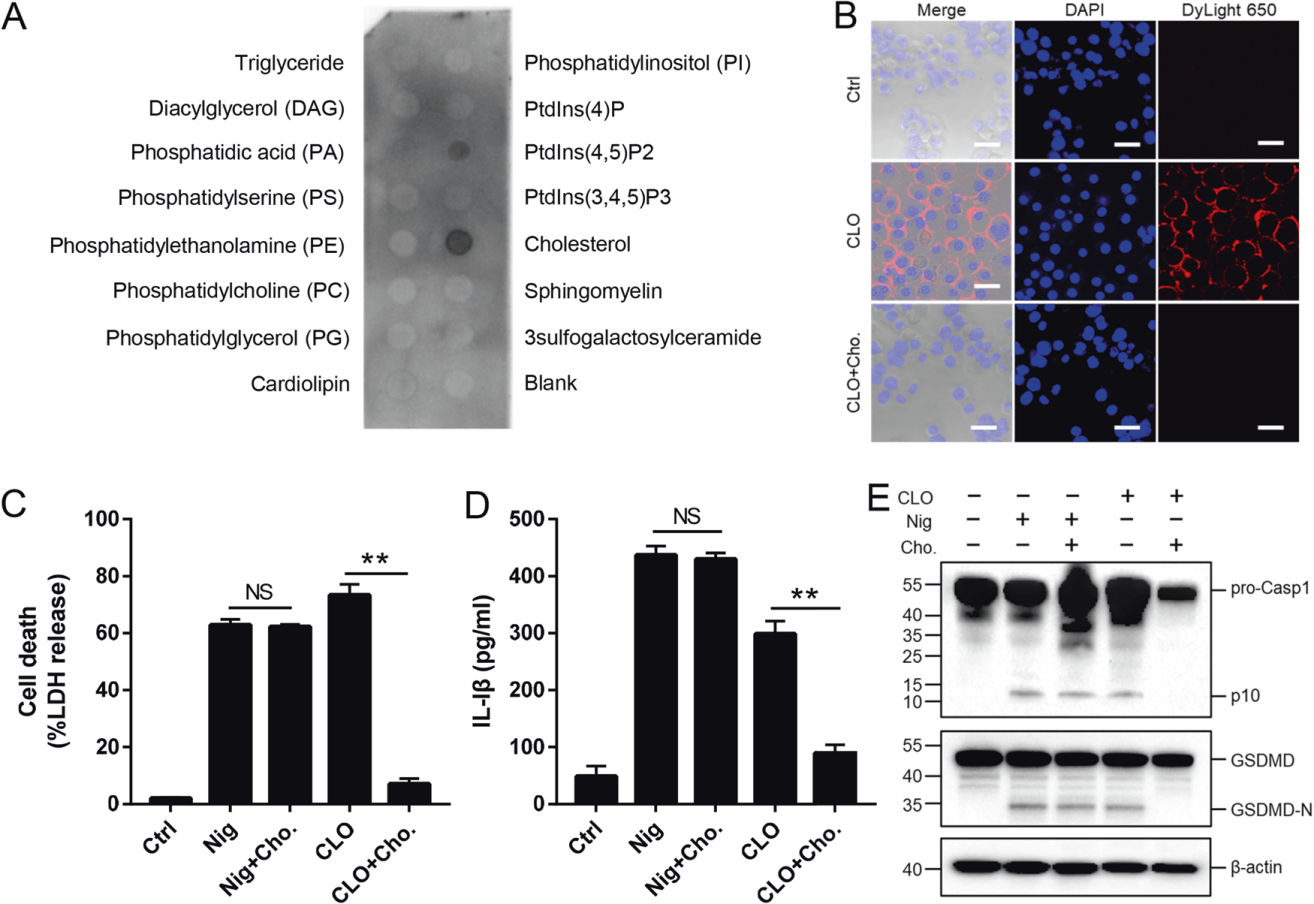
CLO binding to plasma membrane lipids and its effect on CLO-induced pyroptosis. **A** CLO was incubated with a membrane lipid strip spotted with 15 lipids, and the bound CLO was detected by immunoblotting. **B** THP-1 cells were incubated with or without (Ctrl) CLO (100 nM) or cholesterol (Cho.)-pretreated CLO for 1 h. CLO was localized by immunofluorescence microscopy using dyLight 650 anti-6×His tag antibody. Scale bar, 30 μm. **C**–**E** J774A.1 cells were treated with or without (Ctrl) CLO (100 nM), nigericin (Nig), or Cho-pretreated CLO or Nig for 1 h. LDH (**C**) and IL-1β (**D**) release was then determined, and Casp1 and GSDMD cleavage was determined by Western blot with antibodies against Casp1, GSDMD, and β-actin (loading control) (**E**). For panels **C** and **D**, values are shown as means ± SD (*N* = 3). N, the number of replicates. **p < 0.01. NS no significance (student’s unpaired t test).

In general, CDC members are able to interact with plasma cholesterol. In our study, we found that the cytotoxicity of CLO was markedly inhibited in the presence 10% serum but not in the presence of 10% lipid-depleted serum (Supplementary Fig. S5), suggesting that CLO probably interacted with serum lipids, such as cholesterol, which blocked subsequent CLO interaction with the plasma membrane. In support of this hypothesis, lipid binding analysis showed that CLO bound specifically to cholesterol and, to a lesser extent, PtdIns (4,5) P2 (Fig. 5A). Following incubation with J774A.1 cells, CLO was localized on the cellular membrane, and exogenously added cholesterol blocked the membrane binding of CLO (Fig. 5B). Furthermore, the presence of free cholesterol significantly reduced CLO-induced cell death, IL-1β secretion, and Casp1/GSDMD cleavage and activation (Fig. 5C–E).

### CLO cytotoxicity depends on the tryptophan residues in the undecapeptide for membrane binding

**Fig. 6:**
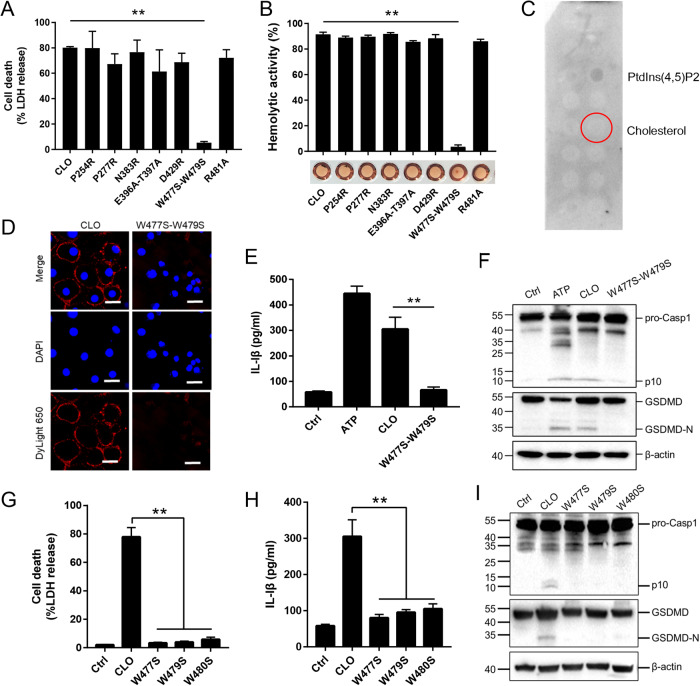
Identification of the functionally important residues of CLO. **A** J774A.1 cells were treated with CLO (100 nM) or its mutants (100 nM) for 1 h, and LDH release was then determined. **B** Sterile defidrinated sheep blood was incubated with CLO (100 nM) or its mutants (100 nM) for 30 min and then detected for hemolysis. **C** A membrane lipid strip was incubated with the W477S-W479S mutant, and the bound protein was detected by immunoblotting. **D** THP-1 cells were incubated with or without (Ctrl) CLO (100 nM) or the W477S-W479S mutant (100 nM) for 1 h. The cells were stained with DAPI and subjected to immunofluorescence microscopy with dyLight 650 anti-6×His tag antibody. Scale bar, 30 μm. **E**, **F** J774A.1 cells were treated with or without (Ctrl) ATP, CLO (100 nM), or the W477S-W479S mutant (100 nM) for 1 h. The cells were determined for IL-1β release (**E**) and Casp1/GSDMD cleavage by immunoblot using antibodies against Casp1, GSDMD, and β-actin (loading control) (**F**). J774A.1 cells were treated with mutants (100 nM) for 1 h. LDH (**G**), IL-1β (**H**) and immunoblot analysis of Casp-1 and GSDMD (**I**) were assessed as above. **p < 0.01. NS no significance (one-way ANOVA test **A**, **B**, **G**, and **H** or student’s unpaired t test **E**. Values are shown as means ± SD (*N* = 3). N the number of replicates.

Like other CDC members, CLO was structurally predicted to contain four domains (D1 to D4), with the D4 domain harboring the undecapeptide (5’- ECTGLAWEWWR-3’) (Supplementary Fig. S6). To find key residues essential to CLO function, seven mutant CLO were created, which bear single residue mutation (P254R, P277R, N383R, D429R and R481A) or double residue mutation (E396A-T397A and W447S-W479S). Compared to CLO, the W477S-W479S mutant exhibited significantly weakened ability to induce cell death and hemolysis, while all other mutants were comparable to CLO in cytotoxicity (Fig. 6A, B). W477S-W479S was unable to bind cholesterol (Fig. 6C) and barely detectable in the cell membrane following incubation with THP-1 cells (Fig. 6D). Consistent with its inability to induce cell death, W477S-W479S triggered no IL-1β release or Casp1/GSDMD cleavage (Fig. 6E, F). Since W477 and W479 are both located in the undecapeptide, the functional importance of these residues, as well as the other tryptophan residue (W480) in the undecapeptide, were further evaluated by creating single residue mutation (W477S, W479S, and W480S). The results showed that mutation of either of these three tryptophan residues deprived CLO of its ability to induce cell death, IL-1β release, and Casp1/GSDMD cleavage and activation (Fig. 6G–I).

## Discussion

Previous reports have indicated that the pathogenicity of *B. cereus* mainly depends on the production of virulence factors. Various toxins, enzymatically active proteases, and phospholipases are closely associated with *B. cereus* diarrheal food poisoning and trigger host immune responses through cytosolic inflammasome sensor or surface Toll like receptor [10, 40–43]. However, the roles of many putative toxins and enzymes, such as the CDC family protein CLO, in *B. cereus* virulence still remain to be explored. In this study, we examined the cytotoxicity of the CLO from a deep-sea *B. cereus* isolate. We found that CLO induced hemolysis and rapid lytic death of mammalian cells at concentrations as low as 1 nM. These results indicated a highly cytotoxic nature of CLO and suggested a role of CLO in the pathogenesis of *B. cereus*.

Among the cytotoxins of *B. cereus*, HBL, NHE, and Cytk are known to be able to activate the NLRP3 inflammasome, whereby linking the toxins to the innate immune inflammasome pathway by establishing a toxin-inflammasome axis [5–7]. Several CDC family members, such as the tetanolysin O of *Clostridium tetani*, the streptolysin O of *Staphylococcus aureus*, and the pyolysin O of *Trueperella pyogenes*, can also activate the NLRP3 inflammasome, and hence are considered to have the potential to trigger the toxin-inflammation pathway [13, 21, 44]. In our study, we found that the toxicity of CLO was blocked by Casp1/NLRP3 inhibitors and under the condition of GSDMD deficiency, but it was not affected by the RIPK or MLKL inhibitors.

Since many NLRP3 activators induce ROS production, ROS is considered to signal NLRP3 inflammasome activation [45–47]. In the present study, significantly elevated ROS was detected in CLO-treated cells, and the presence of ROS inhibitor or scavenger markedly inhibited CLO-induced cell death and Casp1/GSDMD activation. These results indicated a positive contribution of ROS to CLO-mediated NLRP3 activation and downstream pyroptotic signaling. Intracellular K^+^ efflux is another common trigger for NLRP3 inflammasome activation. It has been reported that depletion of intracytoplasmic K^+^ promoted IL-1β maturation and secretion in response to ATP or nigericin, while high extracellular K^+^ blocked the activation of the NLRP3 inflammasome [48, 49]. In the case of CLO, it caused a significant reduction of intracellular K^+^ concentration, and this reduction was required to induce pyroptosis, since supplementation of extracellular K^+^ significantly inhibited CLO-induced Casp1/GSDMD activation and cell death. It was most likely that CLO-induced cell membrane perforation initiated K^+^ efflux, which subsequently activated the NLRP3 inflammasome pathway, resulting in Casp1 and GSDMD-executed pyroptosis. The involvement of the K^+^−NLRP3−Casp1 axis in CLO-induced cell death was further supported by the result that extracellularly added KCL failed to affect the death NLRP3/Casp1-KD cells. However, similar to previous reports, which observed significant but moderate inhibitory effects of extracellularly supplemented KCL on cell death [50, 51], we found that the effect of supplemented KCL on CLO-induced LDH release was, although significant and substantial, relatively moderate (30% reduction), which was possibly due to the fact that CLO could also induce pyroptosis-independent cell death.

The ability of the CDC family proteins to form pores in the cellular membrane is traditionally thought to be absolutely dependent on the interaction with membrane cholesterol [52]. In our study, CLO was observed to bind to cholesterol, and this binding was required for CLO to aggregate in cellular membrane and induce cell death. These observations suggested that cholesterol was the main target for CLO localization in the cell membrane. Recently, eight CDC members were shown to recognize single/multiple glycans as candidate cellular receptors and be able to independently bind glycans and cholesterol [53]. For CLO, we found that in addition to cholesterol, it also bound to PtdIns (4,5) P2. It remains for future studies to examine whether there are other membrane targets involved in bridging CLO to the target cells.

The undecapeptide is the most conserved region in the primary sequence of CDC, and the pore formation capacity of CDC is highly sensitive to changes in the primary structure of the undecapeptide [14, 54, 55]. Substitution of cysteine, tryptophan, or arginine in the undecapeptide motif, such as PFO^R468A^, LLO^C492S^ and PLO^W433F^, rendered CDC proteins incapable of membrane-binding, hemolysis, or cytolysis [54, 56, 57]. One study showed that mutation in any of the tryptophan residues on the undecapeptide of PFO resulted in reduced hemolytic activity but retained cholesterol-binding specificity [58]. For CLO, we observed that the W477S-W479S, but not R481A, mutation disabled CLO to bind cholesterol and plasma membrane, and mutation of either of the three tryptophan residues in the undecapeptide completely abolished cell death and Casp1/GSDMD activation. These results suggested that all of the tryptophan residues were critical for CLO to interact with the membrane target molecules, notably cholesterol, and this interaction was most likely a prerequisite for the oligomerization of CLO molecules to form the pore complexes on the cell membrane. These findings indicated that undecapeptide-mediated binding to the plasma membrane was a prerequisite for *B. cereus* CLO to induce the downstream pyroptosis.

## Materials and methods

### Cell lines

J774A.1 and THP-1 cells were obtained from China Infrastructure of Cell Lines Resource (China). The THP-1 cell line with GSDMD knockout (THP-1 GSDMD-KO) was reported previously [7]. J774A.1 cells were cultured in Dulbecco’s Modified Eagle Medium (DMEM) (Corning, NY, USA) supplemented with 10% (v/v) FBS (Sigma-Aldrich, USA) and 1% penicillin-streptomycin (Yeasen, shanghai, China) at 37 °C with 5% CO_2_. THP-1 and THP-1 GSDMD-KO cells were cultured in RPMI 1640 medium (Gibco, Renfrewshire, UK) supplemented with 10% (v/v) FBS and 1% penicillin and streptomycin. THP-1-Null, THP-1-defCasp1 (Casp1-KD), and THP-1-defNLRP3 (NLRP3-KD) were purchased from InvivoGen and cultured as instructed by the manufacturer.

### Recombinant protein purification

The DNA sequence of CLO (GenBank: CP091971) without signal peptide was amplified by PCR with primers F1 and R1 (Supplementary Table S2). The PCR product was ligated into pET-28a at between the Nde I and Xho I restriction sites using ClonExpress II One Step Cloning Kit (Vazyme, Nanjing, China), yielding pET28a-CLO. The BL21(DE3) *E. coli* strain (Tsingke, Beijing, China) was transformed with pET28a-CLO. The transformant was cultured in LB medium with shaking at 37 °C to OD_600_ 0.6. Isopropyl-β-d-thiogalactopyranoside (ITPG) was added to the culture at the final concentration of 0.06 mM. The culture was continued at 16 °C for 16 h, and then the bacteria were collected by centrifugation (6000 g, 10 min). The bacteria were resuspended in lysis buffer (50 mM NaH_2_PO_4_, 300 mM NaCl, and 10 mM imidazole) and subjected to sonication. The sonicated bacteria were centrifuged (12,000 g, 20 min), and the supernatant was collected and filtered through 0.2 μm filter. The filtered supernatant was purified using nickel-nitrilotriacetic acid (Ni-NTA) columns and eluted with elution buffer (50 mM NaH_2_PO_4_, 300 mM NaCl, and 250 mM imidazole). The eluted proteins were concentrated using Ultra Centrifugal Filter (Millipore, MA, USA). The proteins were separated in 12% SurePAGE gels (GenScript, Nanjing, China) and stained with coomassie blue. The protein concentration was determined using the BCA Protein Assay Kit (GenStar, Beijing, China). To prepare the CLO mutants, the plasmids expressing the mutants (P254R, P277R, N383R, E396A-T397A, D429R, W477S-W479S, R481A, W477S, W479S, and W480S) were constructed by using TransTaq® DNA Polymerase High Fidelity (TransGen Biotech, Beijing, China) to perform inverse PCR with pET28a-CLO as the template and the primer pairs F2/R2-F11/R11, respectively (Supplementary Table S2). Expression and purification of the recombinant CLO mutants were performed as above for CLO.

### Hemolysis assay

The assay was performed as reported previously [59]. For hemolytic activity analysis, CLO variants were adjusted to 1 μM in PBS. The protein (100 μL) was transferred to a U-bottom 96-well microtiter plate in a serial two/ten-fold dilution with PBS. Then, 50 μL of 2% sheep red blood cell (sRBC) suspension was added to each well of the plate, and the plate was incubated at 37 °C for 30 min. For quantitative analysis, 1% sRBC were sonicated on ice or incubated at 37 °C for 30 min, and then centrifuged (1500 g) to collect the supernatant. The OD_450_ of the sonicated sRBC supernatant was defined as 100% hemolysis, while the OD_450_ of the 37 °C-treated sRBC supernatant represented 0% hemolysis. Serially diluted CLO or its mutants were mixed with sRBC (2%) at a 1:1 ratio and incubated at 37 °C for 30 min. The mixture was centrifuged (1500 g), and the supernatant was collected. The supernatant (100 μL) of each sample was transferred to a 96-well microtiter plate, and the absorbance was measured at 450 nm.

### Treatment of cells with CLO

THP-1 cells (as well as variants) were pretreated with phorbol 12-myristate 13-acetate (PMA, 50 nM, Sigma-Aldrich, USA) overnight at 37 °C to differentiate into macrophages before being used for all experiments. J774A.1 and differentiated THP-1 cells were grown overnight to a density of 10^6^ and 5 ×10^5^ cells per well, respectively, in 24-well plates, and then the medium was replaced with Opti-MEM. For J774A.1 cells, the cells were primed with 1 μg/mL LPS for 4 h before CLO treatment. All CLO treatments were performed at 37 °C for 1 h with 100 nM CLO/variant unless otherwise stated. To examine the effect of necroptosis and pyroptosis inhibitors, the cells were treated with GSK’963 (5 µM, Selleck, TX, USA), GSK’872 (3 µM, Selleck), GW806742X (5 µM, MCE, NJ, USA), Q-VD-OPh (5 µM, MCE), Ac-YVAD-CMK (50 µM, MCE), Ac-DEVD-CHO (5 µM, MCE), Z-LEVD-FMK (50 µM, MCE), Z-IETD-FMK (5 µM, MCE), MCC950 (50 µM, Selleck), or VX-765 (50 µM, Selleck) at 37 °C for 1 h. The cells were then treated with or without CLO. To examine the effect of ROS inhibitors, the cells were treated with DPI (20 μM, Selleck), or NAC (20 mM, Selleck) at 37 °C for 1 h before CLO (10 nM) treatment. To examine the effect of cholesterol and K^+^, the cells were treated with cholesterol (100 μg/ml) and various concentrations of KCl (6.25–100 mM) for 30 min and then treated with CLO. As a positive control of pyroptosis, nigericin (20 μM, Sigma-Aldrich) or ATP (5 mM, Sigma-Aldrich) was incubated with the cells at 37 °C for 1 h. To induce necroptosis, THP-1 cells were treated with a combination of TNFα (30 ng/ml), the SMAC mimetic BV-6 (1 mM) and Z-VAD-FMK (50 µM) for 16 h at 37 °C.

### Immunoblot

The cell culture supernatants or cell lysates were prepared as reported previously [7]. For immunoblotting, the samples were mixed with SDS-PAGE buffer and heated at 100 °C for 10 min, followed by electrophoresis in 12% polyacrylamide gel (GenScript). The proteins were transferred to a nitrocellulose membrane. The membrane was blocked with 5% skimmed milk for 1 h at room temperature and then incubated overnight with the primary antibodies against caspase-1 (1:1000 dilution) (Abcam, Cambridge, MA, USA) and GSDMD (1:1000 dilution) (CST, MA, USA). Next, the membrane was incubated with HRP goat-anti-rabbit (1:2000 dilution) (ABclonal, Wuhan, China) for 1 h. The immunoreactive proteins were detected using ECL plus kit (Shandong Sparkjade Biotechnology Co., Ltd, China) and imaged with the GelDoc XR System (Bio-Rad, PA, USA).

### Immunofluorescence microscopic analysis

J774A.1 cells or THP-1 cells were transferred to glass-bottomed dishes (NEST Biotechnology, Wuxi, China) and incubated overnight to 10^6^ or 5 ×10^5^ cells/well. The cells were treated with CLO variants as described above for 1 h, washed three times with PBS, and fixed with 4% paraformaldehyde at room temperature for 15 min, followed by blocking in 5% BSA in PBST (PBS with 0.1% Tween 20) for 1 h. To examine membrane binding, the cells were incubated with DyLight® 650 Anti-6 × His tag antibody (1:200 dilution, Abcam) for 1 h at room temperature. To examine ASC speck formation, the cells were incubated with ASC-antibody (1:1000, Abclonal) for 1 h and then incubated with anti-Rabbit IgG antibody conjugated with Alexa Fluor 594 (1:200, Abcam) for 1 h. Cells were washed as above with PBST and incubated with DAPI (Sangon, Shanghai, China) for 15 min. The cells were observed with a confocal microscope (Carl Zeiss LSM710, Jena, Germany).

### Cell viability, cytotoxicity and IL-1β release assay

Cell viability was assessed using Calcein AM Cell Viability Assay Kit (Beyotime) according to the manufacturer’s instructions. The cytoplasmic lactate dehydrogenase (LDH) released by the treated cells was evaluated using the CytoTox 96® Non-Radioactive Cytotoxicity Assay kit (Promega, Leiden, Netherlands). IL-1β release was measured using mouse IL-1β ELISA kit (Solarbio, Beijing, China) and human IL-1β ELISA kit (Solarbio) according to the manufacturer’s instructions.

### Protein-lipid binding assay

The binding of CLO to lipids was determined using Membrane Lipid Strips (Echelon Biosciences, UT, USA) as reported previously [60]. The lipid strips were pretreated in Buffer A (PBST containing 3% BSA) for 1 h at room temperature and then covered with CLO in Buffer A for 1 h. The lipid strips were washed 3 times with PBST and incubated with HRP-conjugated Mouse anti-His-Tag antibody (1:1000 dilution, ABclonal) in Buffer A for 1 h. The lipid strips were detected using ECL kit (Sparkjade) and imaged with the GelDoc XR System.

### Intracellular K+ detection

J774A.1 cells were cultured overnight to 2 × 10^6^ cells/well in 12-well plates as described above. The cells were treated with CLO (100 nM) or Nig (20 μM) for 30 minutes in fresh Opti-MEM medium and then washed three times with potassium-free PBS (NaCl, 140 mM; Na_2_HPO_4_, 10 mM; NaH_2_PO_4_, 2 mM). The cells were treated with concentrated nitric acid to obtain cell lysates, and then the concentration of intracellular K^+^ was measured by inductively coupled plasma optical emission spectrometry (ICP-OES) with a PerkinElmer Optima 7300 DV spectrometer.

### Statistical analysis

Statistical analysis was carried out using GraphPad Prism 7.0 (GraphPad, San Diego, CA, USA). Statistical significance was determined with Student’s *t* test for two groups or with one-way analysis of variance (ANOVA) for more than two groups. *P* < 0.05 was considered statistically significant.

### Supplementary information

Table S1 and S2; Figure S1 to S6

Original Data File

## Data Availability

All data in the paper are present in the paper or the Supplementary Materials.
