# Supplementary material for: Bacillus cereus cereolysin O induces pyroptosis in an undecapeptide-dependent manner
Source: Cell Death Discov. 2024 Mar 8;10:122. doi: 10.1038/s41420-024-01887-7 (PMC10923922; doi:10.1038/s41420-024-01887-7)

**Supporting information**

**Table S1. The sequence comparison of CLO from MB1 and ten human clinical isolates of the *Bacillus cereus* group (from Pathogen Detection of NCBI)**

| Strain | Isolation type | Host | BioSample | Assembly | GenBank | RefSeq | Identity  (amino acid) |
| --- | --- | --- | --- | --- | --- | --- | --- |
| B4264 | clinical | Homo sapiens | SAMN02604059 | GCA_000021205.1 | CP001176.1 | NC_011725.1 | 99.61 |
| H9401 | clinical | Homo sapiens | SAMN02603474 | GCA_000258885.1 | CP002091.1 | NC_017729.1 | 95.7 |
| 97-27 | clinical | Homo sapiens | SAMN03223175 | GCA_000833085.1 | CP010088.1 | NZ_CP010088.1 | 96.29 |
| FDAARGOS_235 | clinical | Homo sapiens | SAMN04875572 | GCA_002073415.2 | CP020437.2 | NZ_CP020437.2 | 91.99 |
| PNO2 | clinical | Homo sapiens | SAMN25416578 | GCA_022014755.1 | CP091762.1 | NZ_CP091762.1 | 95.9 |
| ChBA30D | clinical | Homo sapiens | SAMN25419190 | GCA_022014795.1 | CP091767.1 | NZ_CP091767.1 | 95.9 |
| A178 | clinical | Homo sapiens | SAMN13164747 | GCA_022220985.1 | CP076173.1 | NZ_CP076173.1 | 95.9 |
| A27 | clinical | Homo sapiens | SAMN13164726 | GCA_022221045.1 | CP076181.1 | NZ_CP076181.1 | 95.9 |
| UR-1 | clinical | Homo sapiens | SAMN18241472 | GCA_022221125.1 | CP076192.1 | NZ_CP076192.1 | 95.9 |
| MV19 | clinical | Homo sapiens | SAMN28870333 | GCA_023824275.1 | CP098734.1 | NZ_CP098734.1 | 99.8 |

**Table S2. Primers used in this study.**

| Primer | Sequence |  |
| --- | --- | --- |
| F1 (CLO) | GAAACACAAGCAAGTAATGC |  |
| R1 (CLO) | ATGACTAATACTAGCTGTTG |  |
| F2 (P254R) | GTGCTGAACTACCTAACAATCGATCCGACCTTTTTGAT |  |
| R2 (P254R) | ATCAAAAAGGTCGGATCGATTGTTAGGTAGTTCAGCAC | |
| F3 (P277R) | GCGTAAGTAATTCGGCTCGCCCTGTTATGGTTTCAAATG |  |
| R3 (P277R) | CATTTGAAACCATAACAGGGCGAGCCGAATTACTTACGC |  |
| F4 (N383R) | AAGTACTTTCTTAAAAGATAGGGCAACCGCTGCTGTT |  |
| R4 (N383R) | AACAGCAGCGGTTGCCCTATCTTTTAAGAAAGTACTT |  |
| F5 (E396A-T397A) | CAATACAGATTATATTGCGGCGACAACTACAGAATATTC |  |
| R5 (E396A-T397A) | GAATATTCTGTAGTTGTCGCCGCAATATAATCTGTATTG |  |
| F6 (D429R) | CTTGGGATGAATTCACATTTCGCCAAAAGGGTAACGA |  |
| R6 (D429R) | TCGTTACCCTTTTGGCGAAATGTGAATTCATCCCAAG |  |
| F7 (W477S-W479S) | GTACAGGTCTTGCAAGTGAAAGCTGGAGAACAATTA |  |
| R7 (W477S-W479S) | TAATTGTTCTCCAGCTTTCACTTGCAAGACCTGTAC |  |
| F8 (R481A) | CTTGCATGGGAATGGTGGGCAACAATTATTAATGAAC |  |
| R8 (R481A) | GTTCATTAATAATTGTTGCCCACCATTCCCATGCAAG |  |
| F9 (W477S) | GAGAATGTACAGGTCTTGCAAGCGAATGGTGGAGAAC |  |
| R9 (W477S) | GTTCTCCACCATTCGCTTGCAAGACCTGTACATTCTC |  |
| F10 (W479S) | CAGGTCTTGCATGGGAAAGCTGGAGAACAAT |  |
| R10 (W479S) | ATTGTTCTCCAGCTTTCCCATGCAAGACCTG |  |
| F11 (W480S) | CAGGTCTTGCATGGGAATGGAGCAGAACAATTATTAATGAAC |  |
| R11 (W480S) | GTTCATTAATAATTGTTCTGCTCCATTCCCATGCAAGACCTG |  |

**Figure S1**. SDS-PAGE analysis of CLO and its mutants. Purified recombinant CLO (A) and mutants (B) were analyzed by SDS-PAGE and viewed after staining with Coomassie brilliant blue R-250.


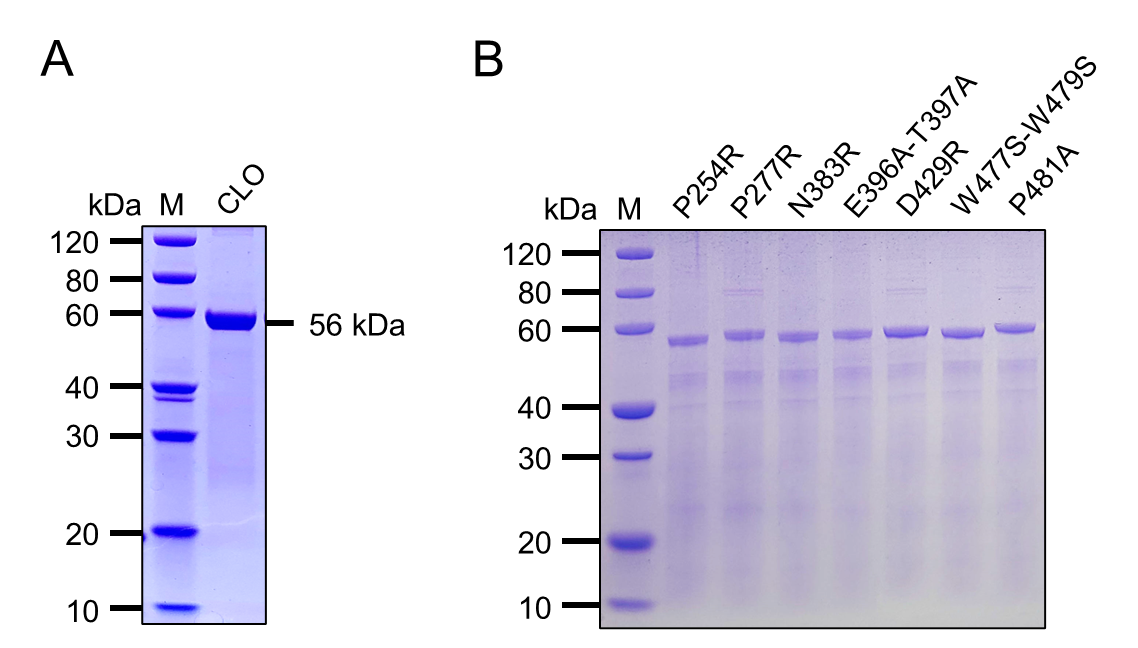


**Figure S2**. The cytotoxic effect of CLO on J774A.1 cells. J774A.1 cells were incubated with or without (Ctrl) CLO at different doses for 1 h and then measured for LDH release. The values are shown as means ± SD (N = 3). N, the number of replicates. ** p < 0.01.


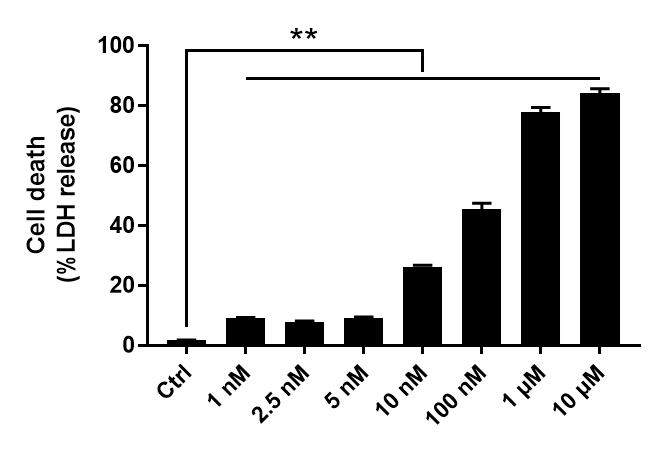


**Figure S3**. The involvement of caspase 1 and GSDMD in CLO-induced cell death. (A) J774A.1 cells were treated with or without (Ctrl) CLO (100 nM) in the presence of Q-VD-OPh, Ac-YVAD-CMK, Ac-DEVD-CMK, Z-IETD-FMK, or DMSO for 1h. LDH release was then measured. (B) J774A.1 cells were treated with or without (Ctrl) CLO (100 nM) in the presence or absence of different concentrations of Ac-YVAD-CMK for 1 h. LDH release was then measured. (C) THP-1 Null, THP-1 Casp1-KD and THP-1 GSDMD-KO cells were incubated with or without (Ctrl) CLO (100 nM) for different time and then measured for LDH release. For panels A and B, values are shown as means ± SD (N = 3). N, the number of replicates. ** p < 0.01, * p < 0.05. NS, no significance.


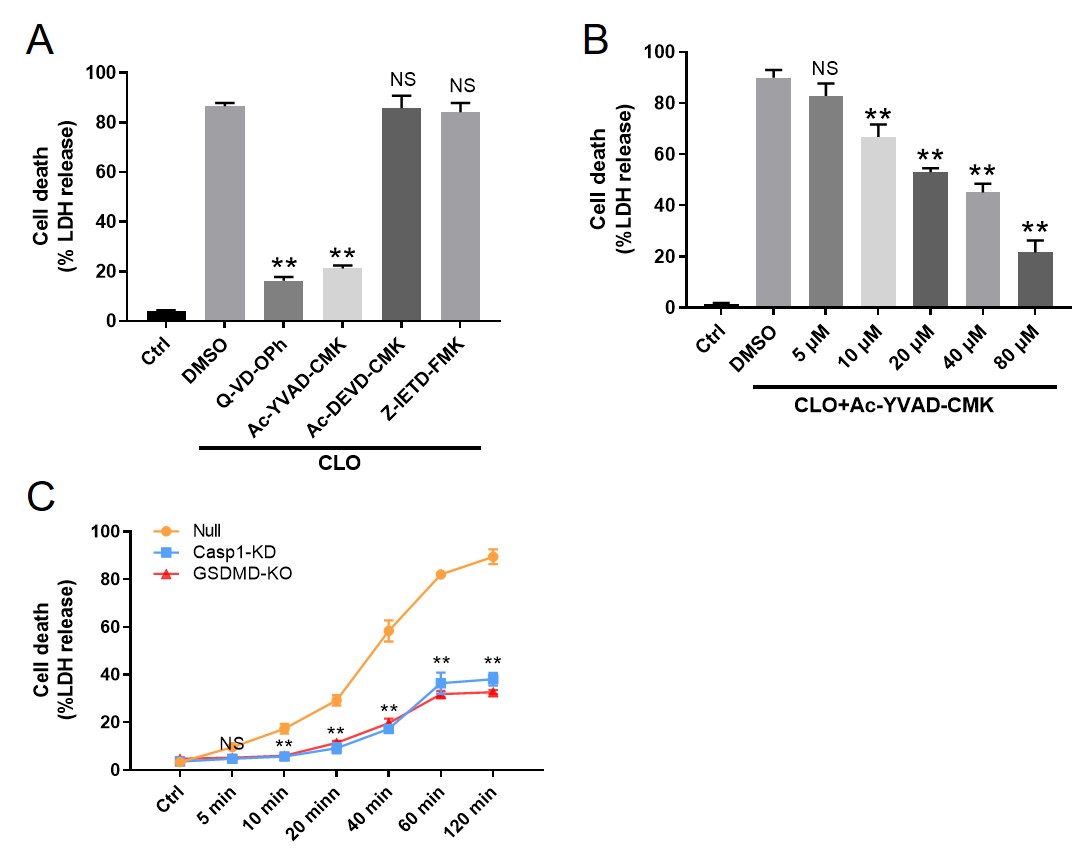


**Figure S4**. The involvement of ROS production and K^+^ efflux in CLO-induced pyroptosis. (A) J774A.1 cells were pretreated with or without (−) DPI or NAC for 1 h and then treated with CLO (100 nM) or ATP for 1 h. LDH and IL-1β releases were then determined. (B, C) PMA-differentiated THP-1 cells with or without (Null) deficiency in NLRP3 (NLRP3-KD) or Casp1 (Casp1-KD) were pretreated with or without (−) different concentrations of KCl for 1 h, and then treated with or without (−) CLO (100 nM) for 1 h. LDH and IL-1β releases were then determined (B). Casp1 and GSDMD cleavage was determined by Western blot with antibodies against Casp1, GSDMD, and β-actin (loading control) (C). For panels A and B, values are shown as means ± SD (N = 3). N, the number of replicates. ** p < 0.01. NS, no significance.


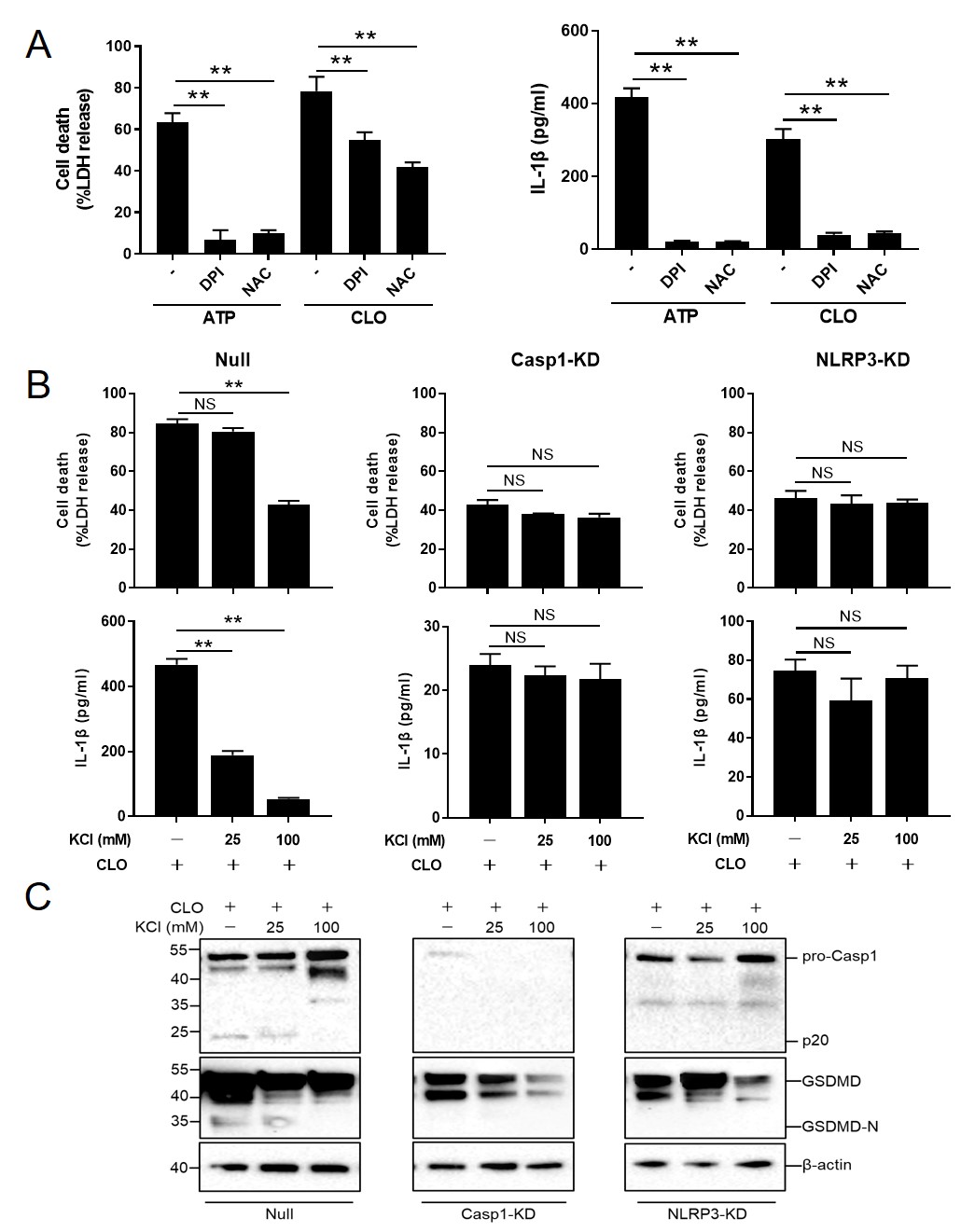


**Figure S5.** J774A.1 cells were incubated with CLO in DMEM supplemented with or without 10% FBS, or 10% lipid-depleted FBS (LD-FBS) for 60 min, and then observed with a microscope. Scale bar, 10 μm.


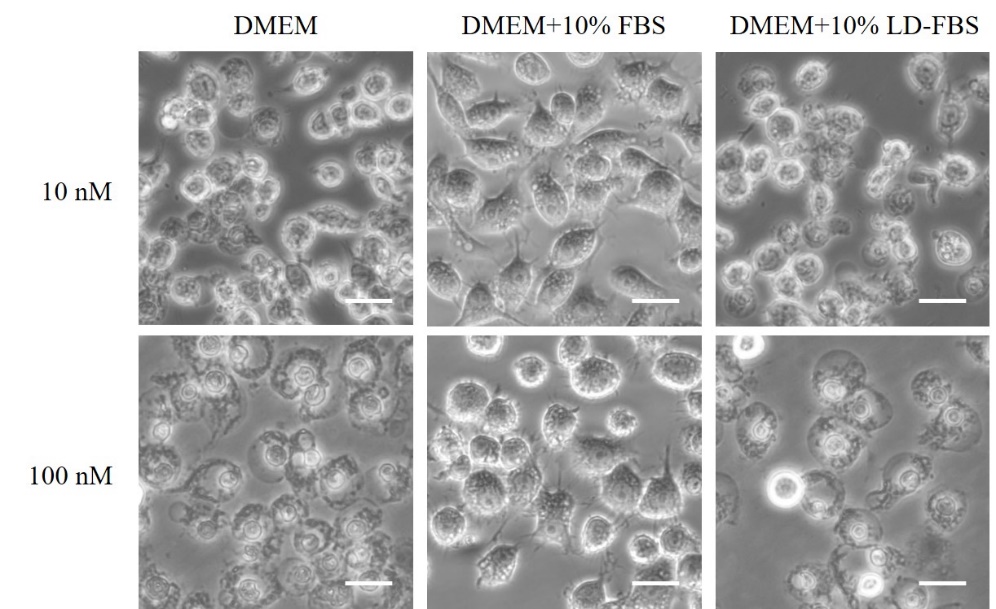


**Figure S6.** The predicted structure of CLO. The mutated amino acid residues are shown in red (P254), green (P277), yellow (N383), pink (E397T397), blue (D429), orange (W477, W479 and W480), and purple (R481).


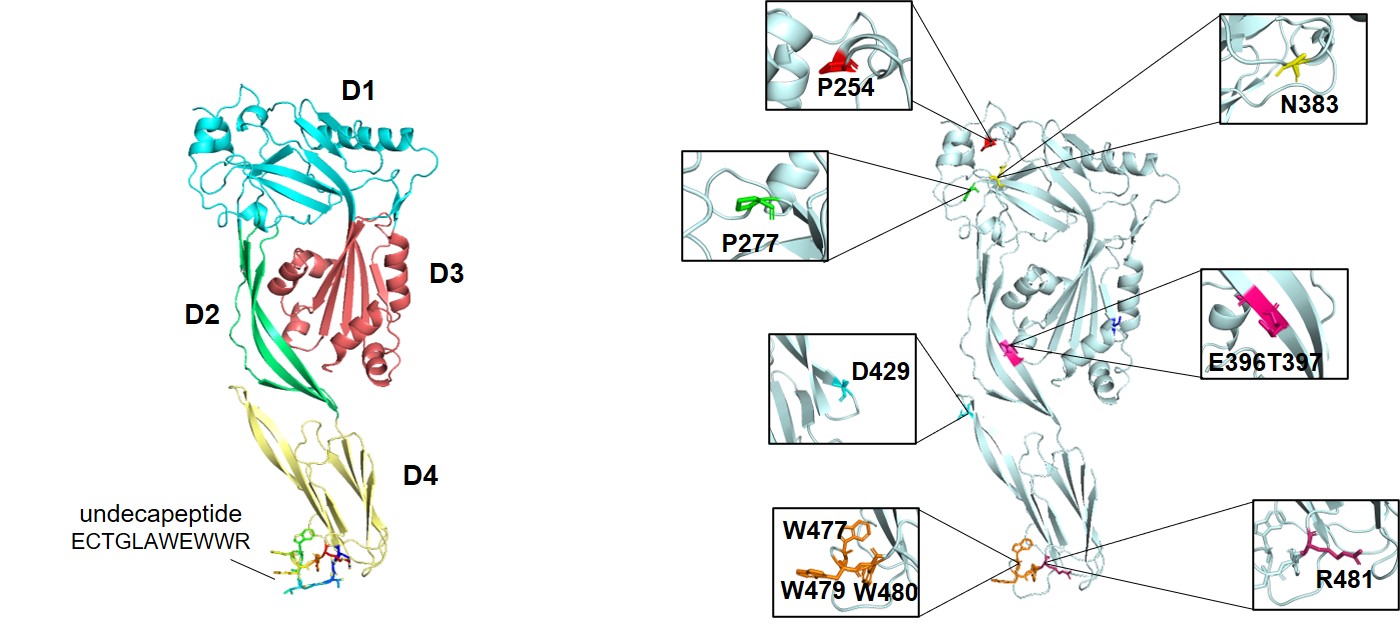

Supplement: Supplementary file 1 — Table S1 and S2; Figure S1 to S6 [file 41420_2024_1887_MOESM1_ESM.docx]
