# Supplementary figures and images for: Bacillus cereus cereolysin O induces pyroptosis in an undecapeptide-dependent manner
Source: Cell Death Discov. 2024 Mar 8;10:122. doi: 10.1038/s41420-024-01887-7 (PMC10923922; doi:10.1038/s41420-024-01887-7)

**Figure 3B and 3C**

**
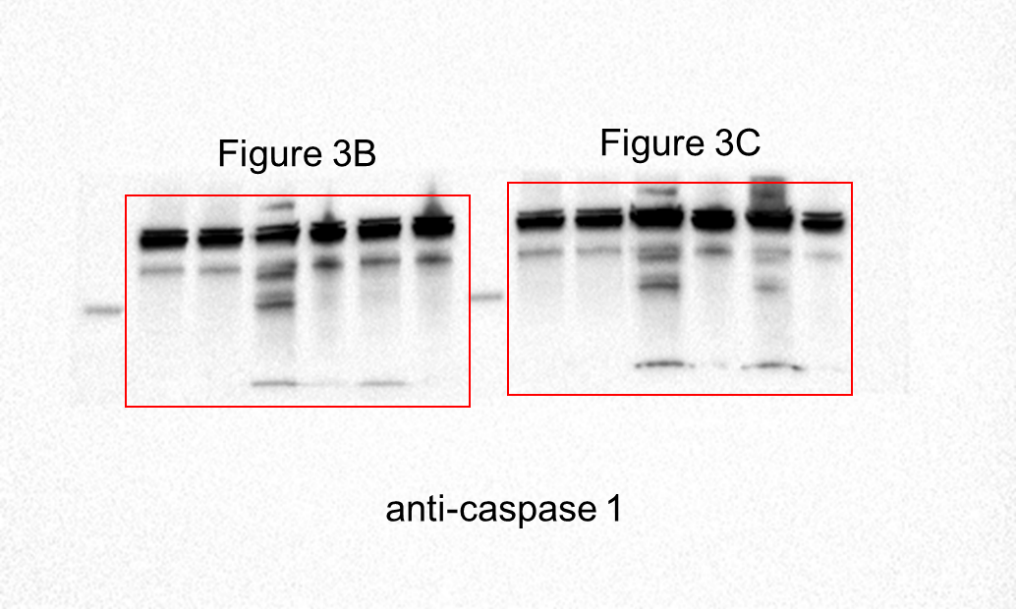
**


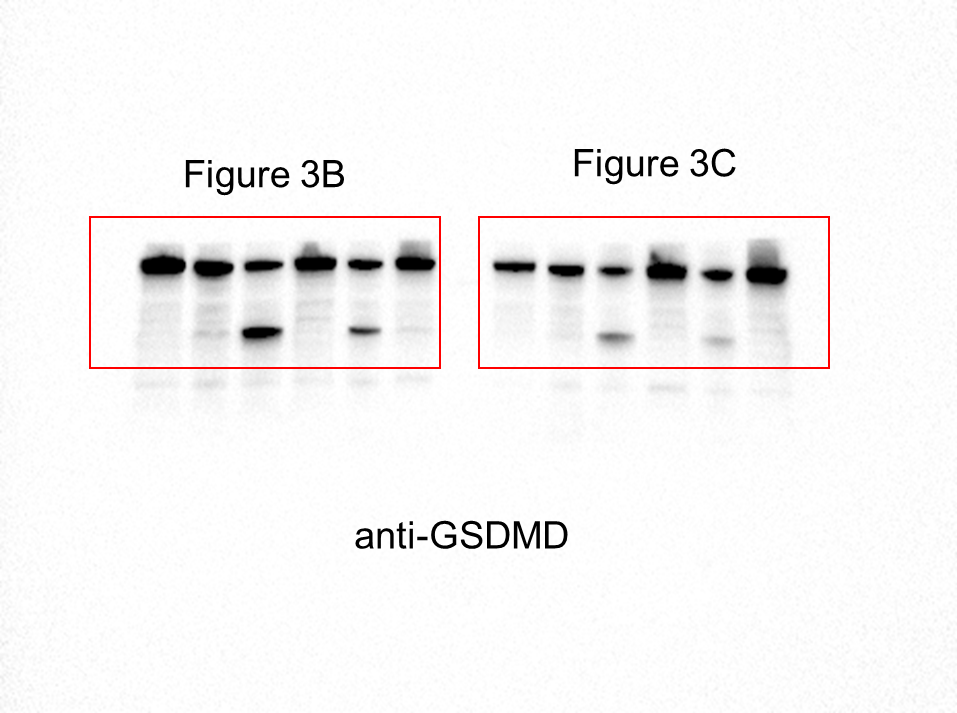


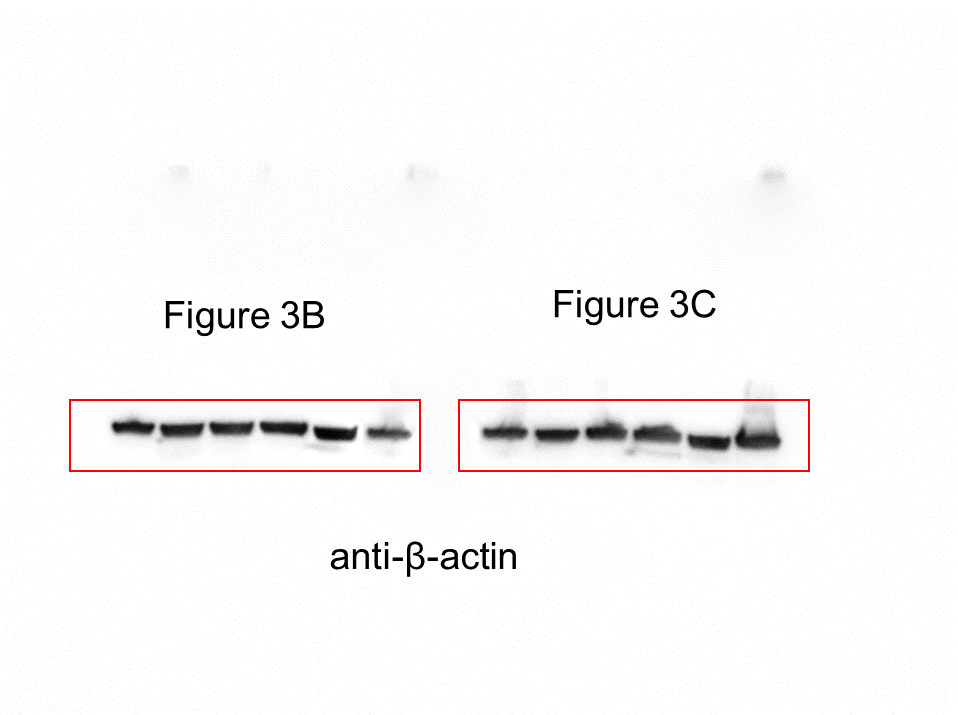


**Figure 3F**


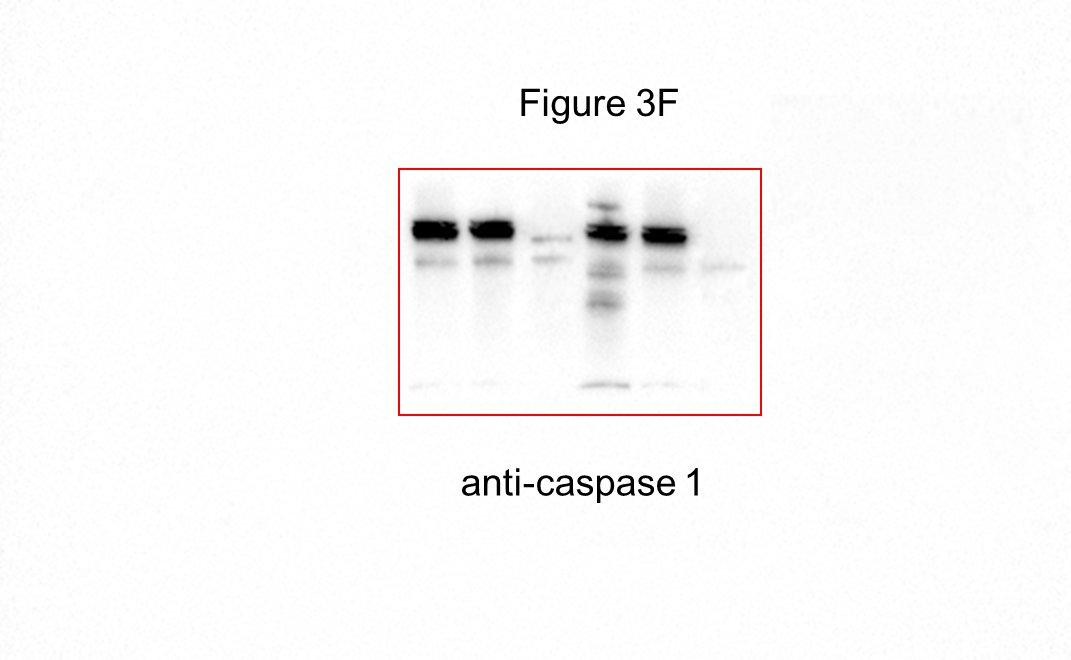


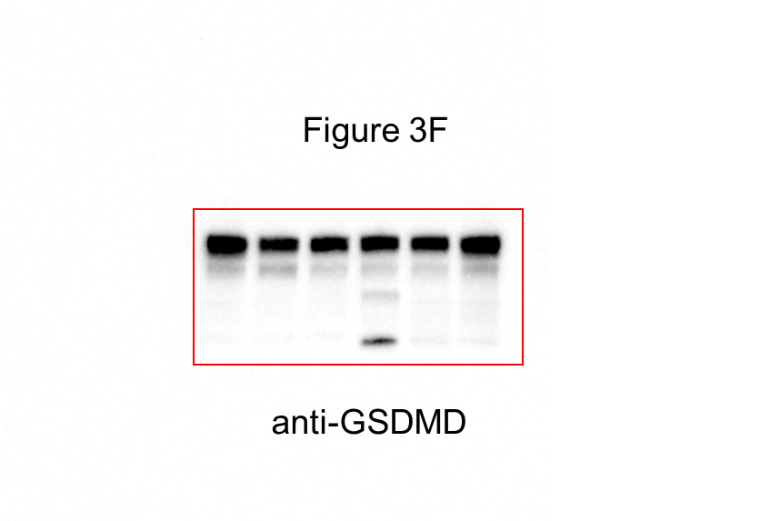


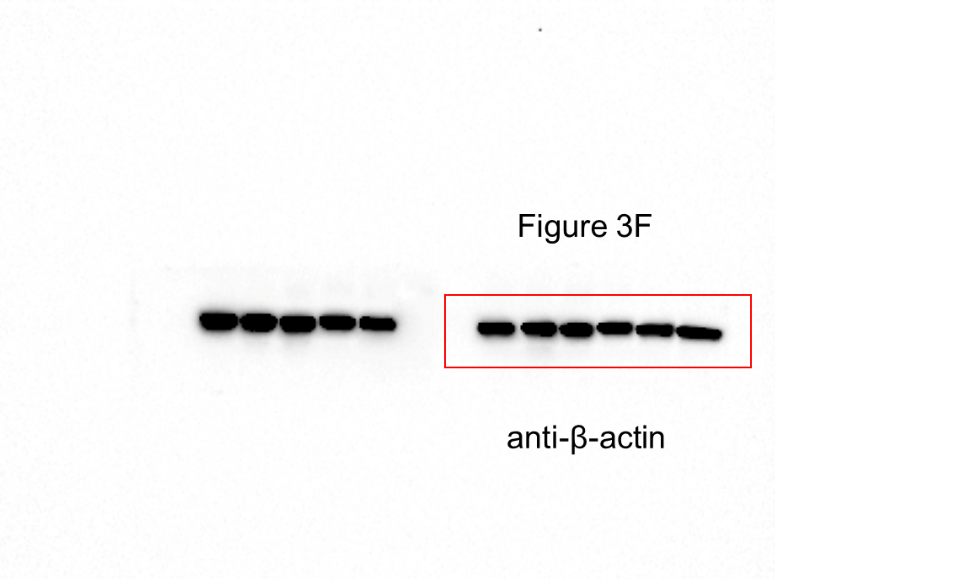


**Figure 4G**


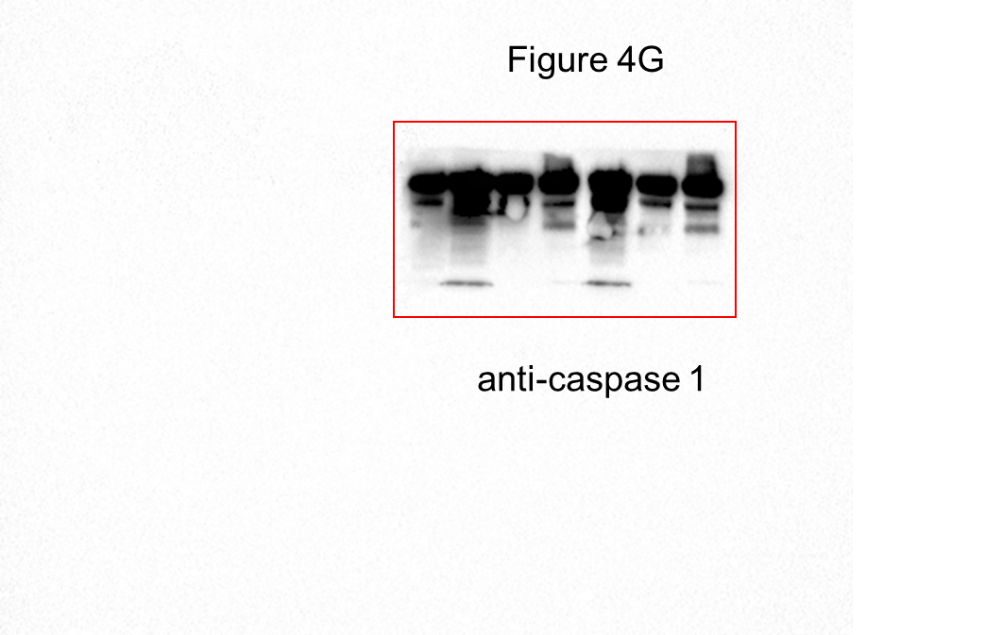


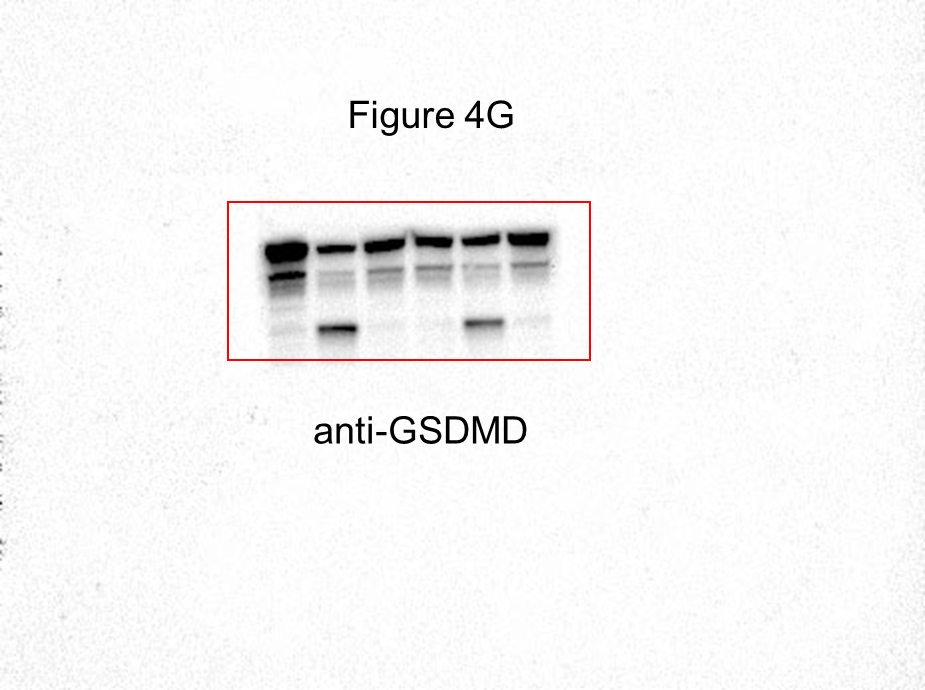


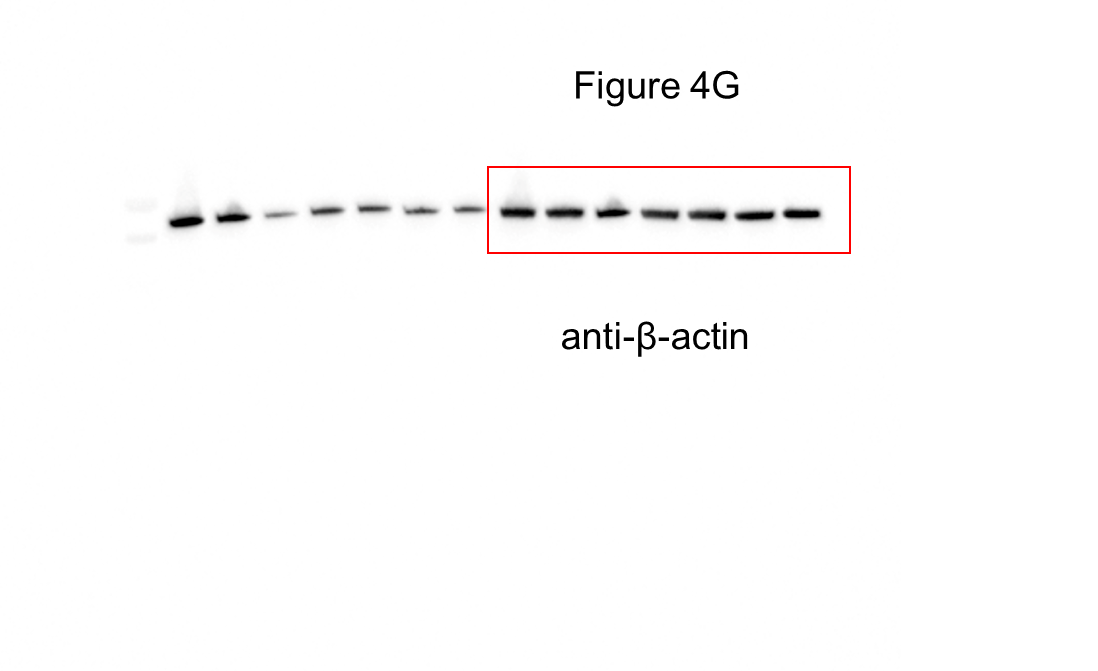


**Figure 4K**


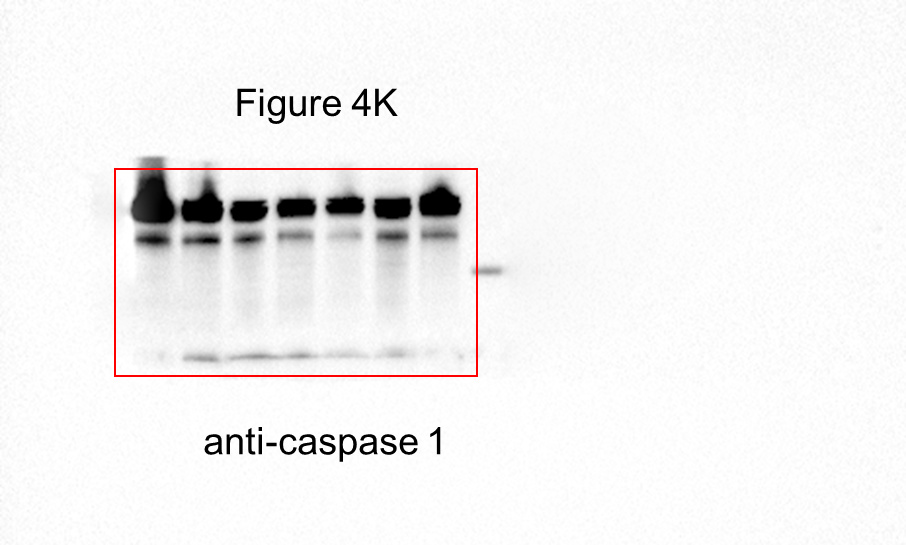


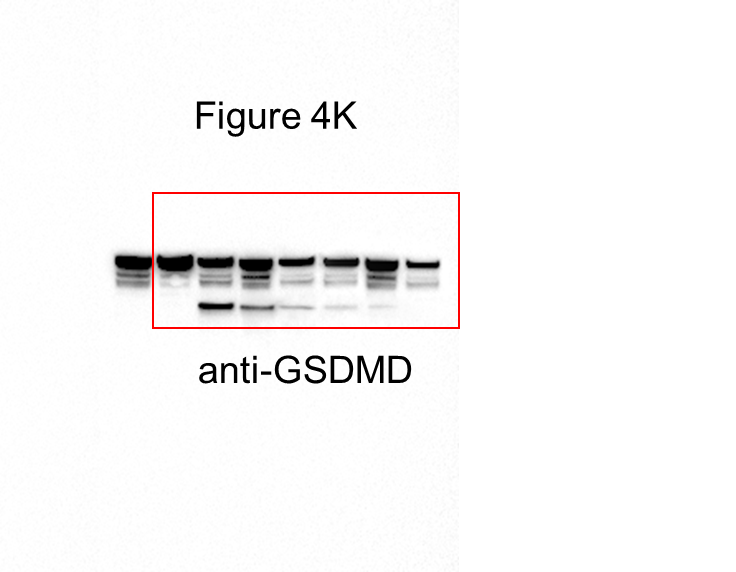


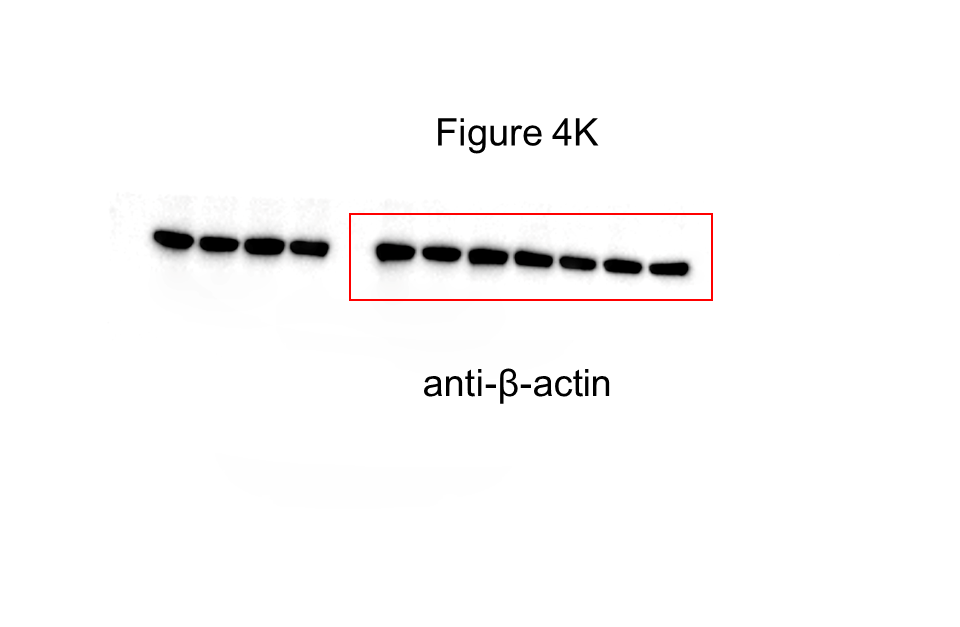


**Figure 5A**


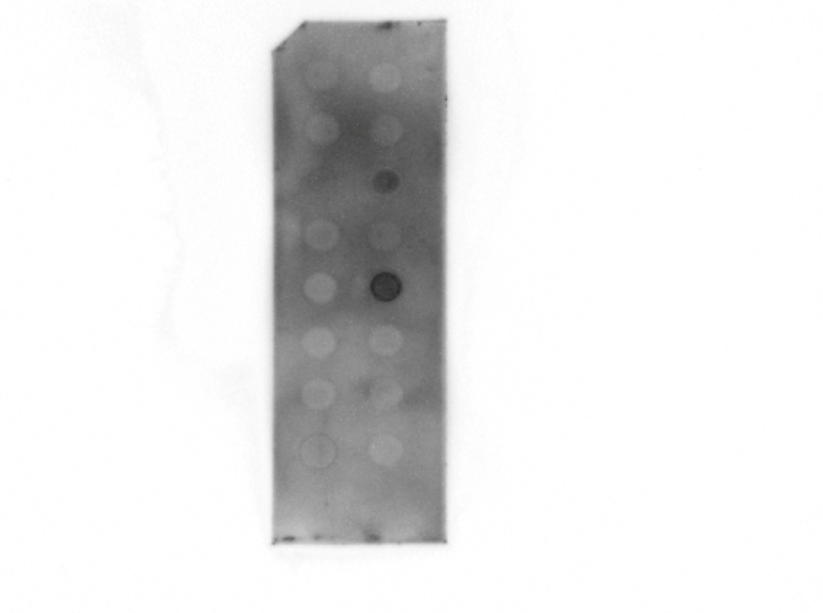


**Figure 5E**


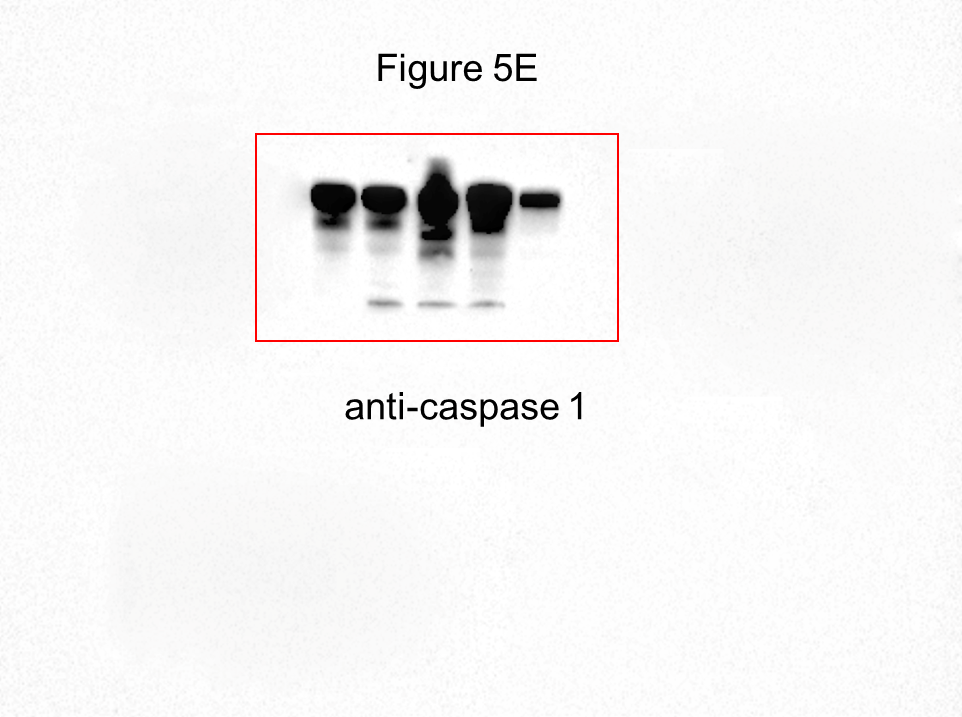


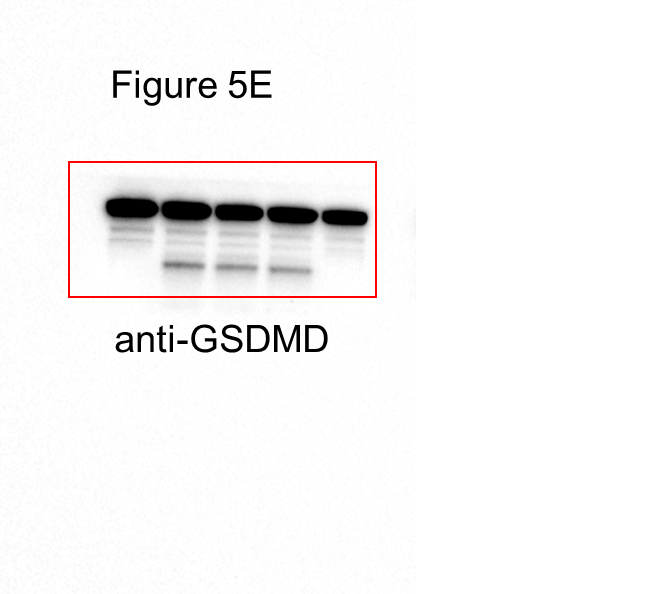


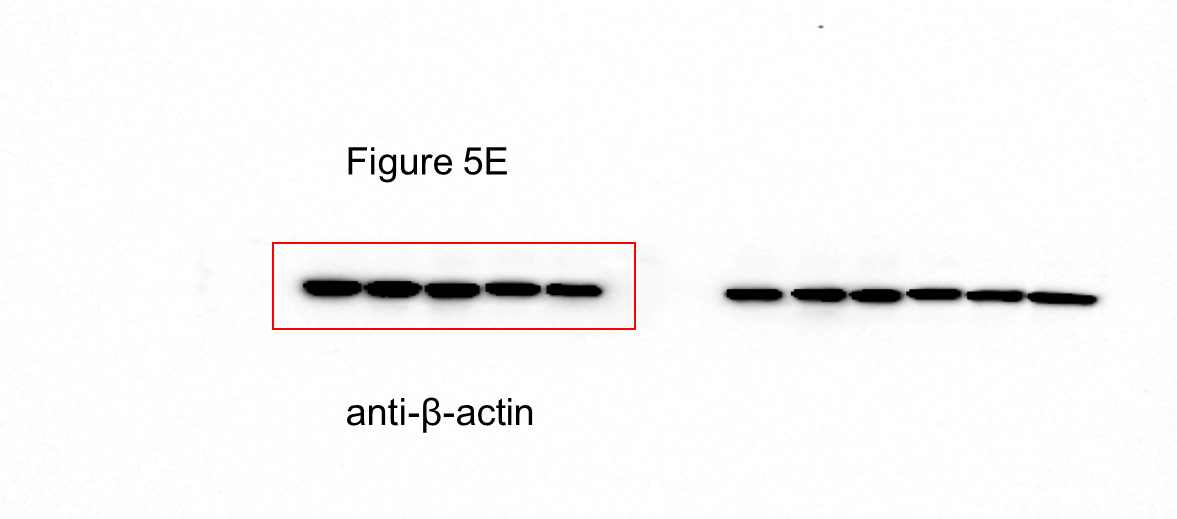


**Figure 6C**


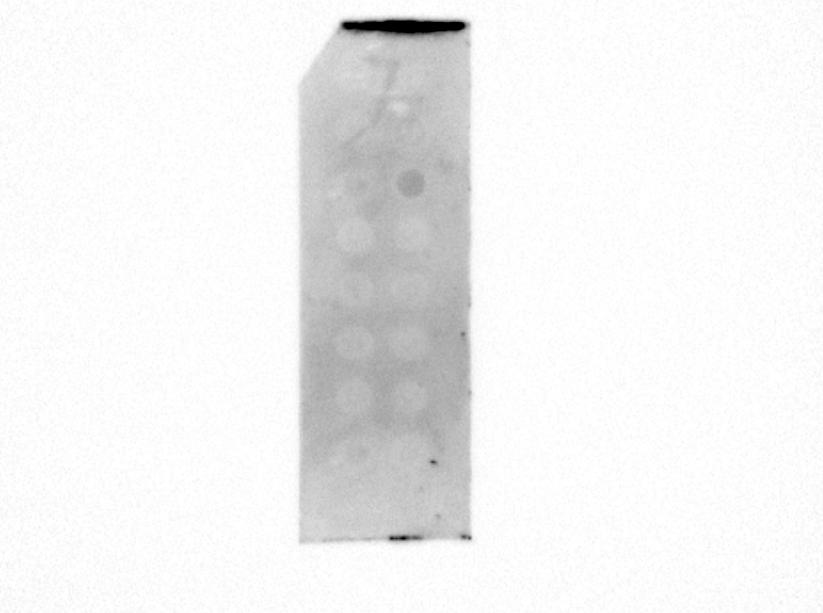


**Figure 6F**


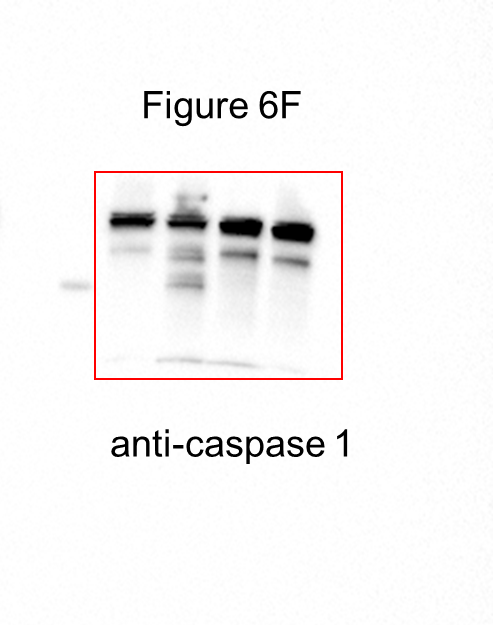


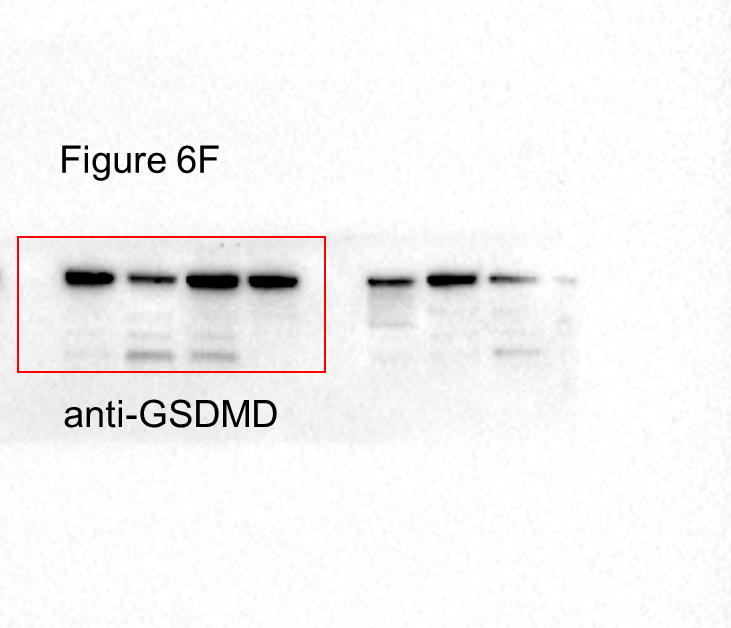


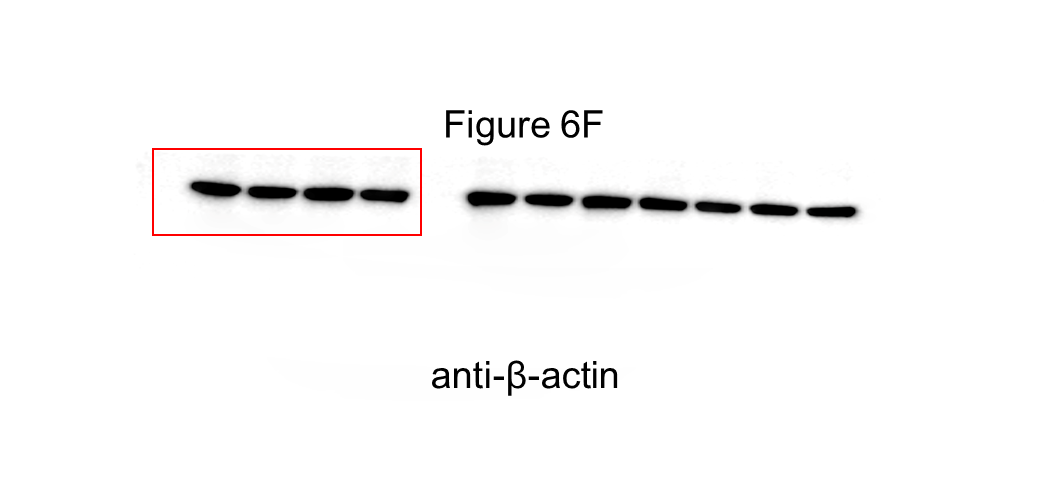


**Figure 6I**


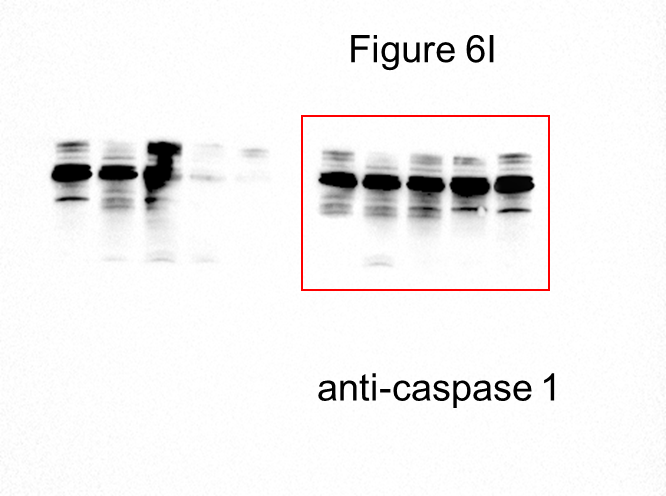


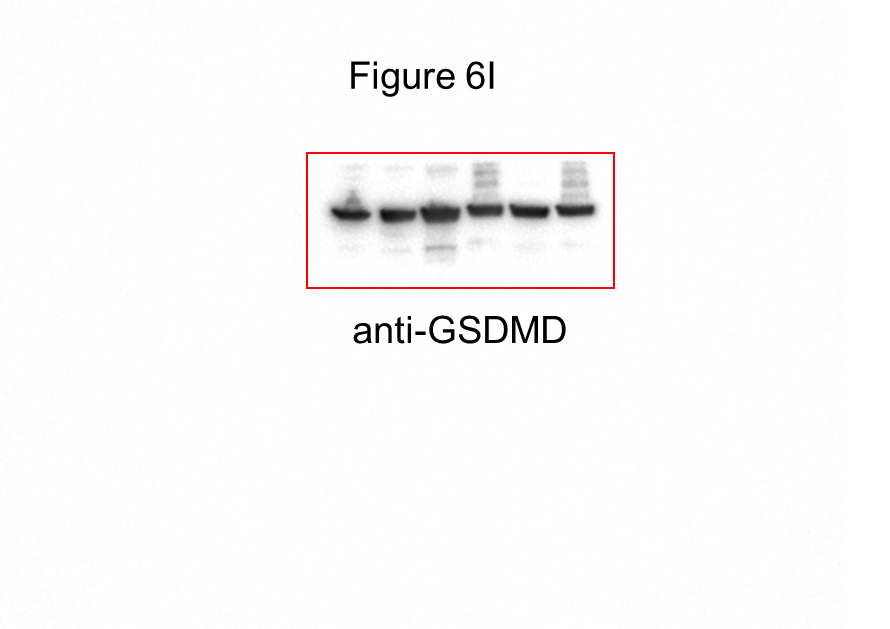


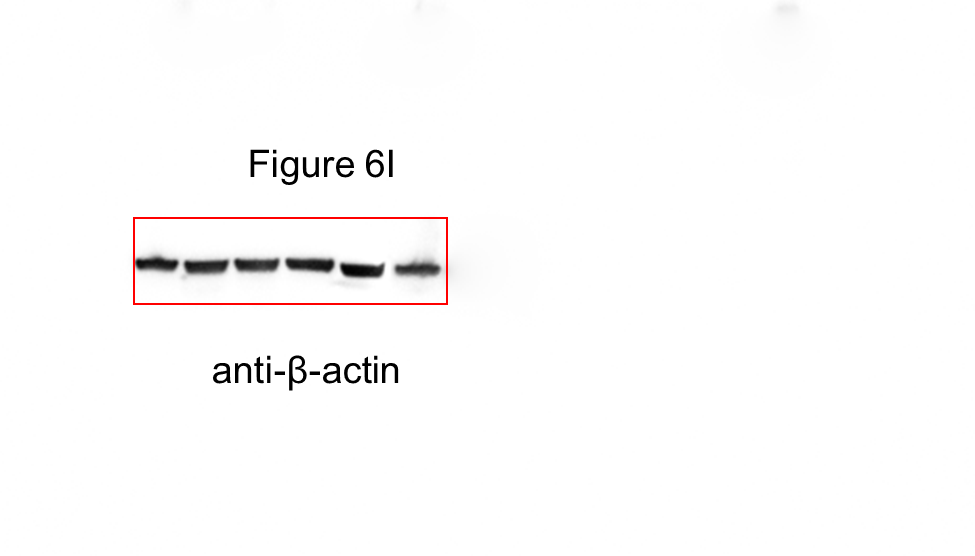


**Figure S4C**


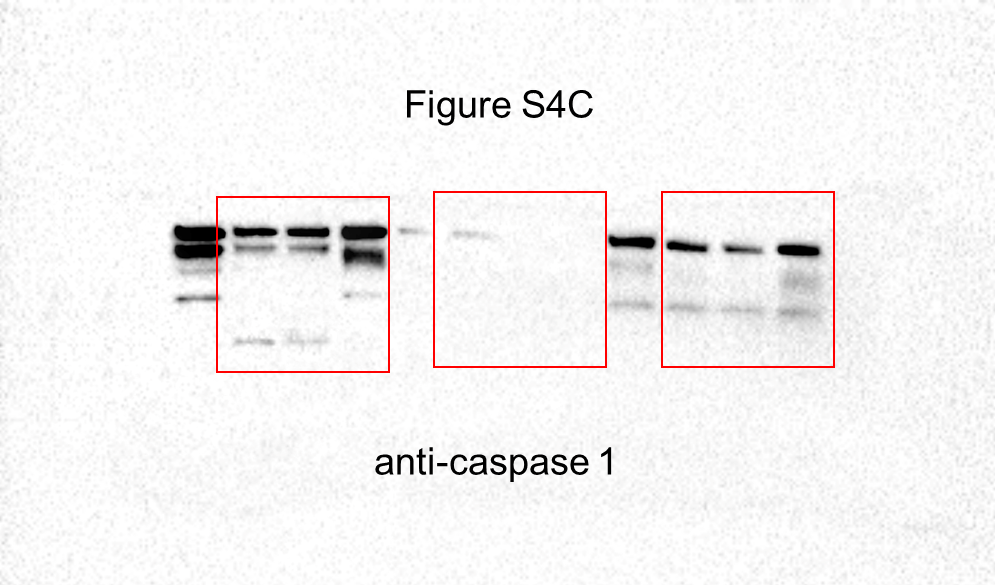


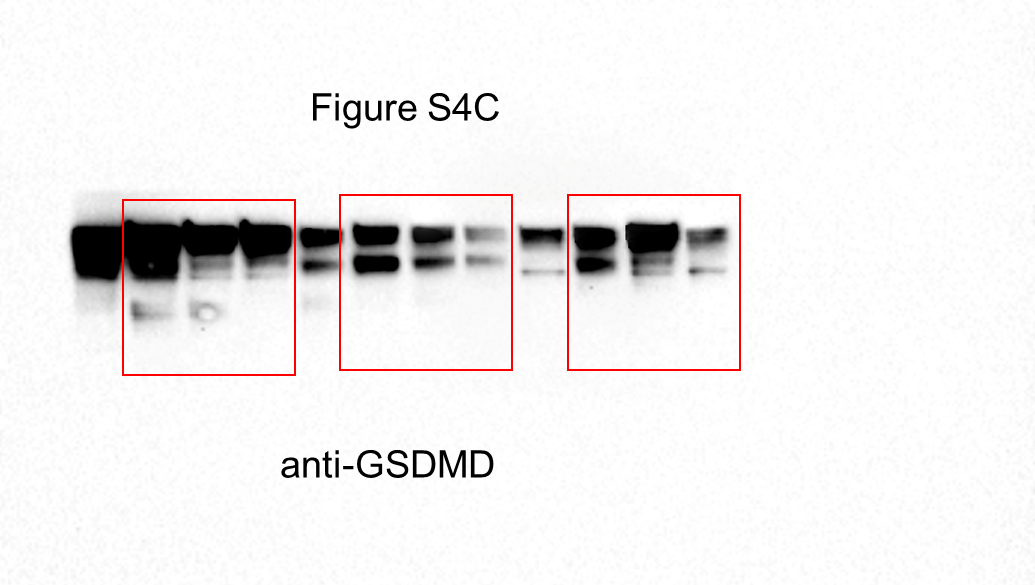


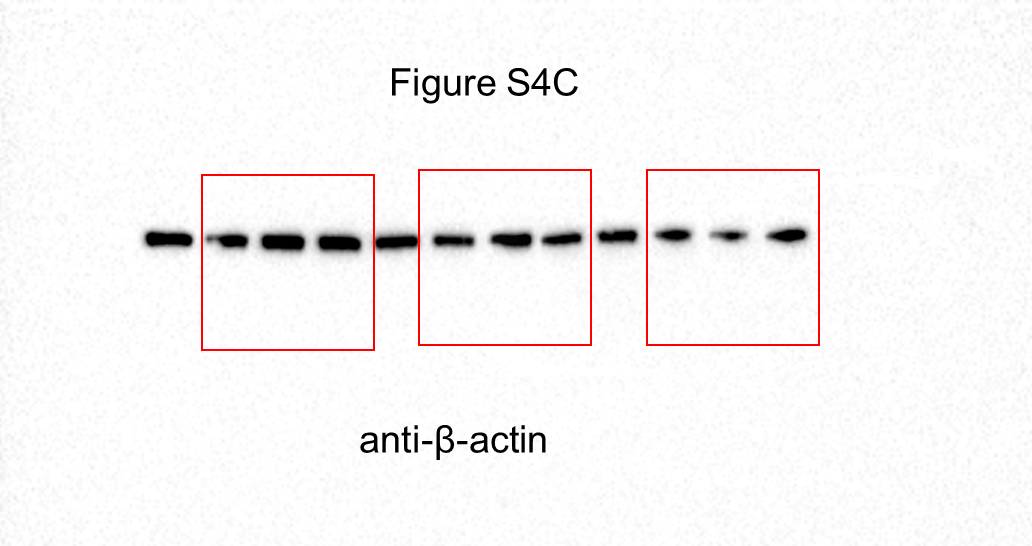

Supplement: Supplementary file 2 — Original Data File [file 41420_2024_1887_MOESM2_ESM.docx]
